# Proteome response of *Phaeodactylum tricornutum*, during lipid accumulation induced by nitrogen depletion

**DOI:** 10.1016/j.algal.2016.06.015

**Published:** 2016-09

**Authors:** Joseph Longworth, Danying Wu, María Huete-Ortega, Phillip C. Wright, Seetharaman Vaidyanathan

**Affiliations:** ChELSI Institute, Advanced Biomanufacturing Centre, Department of Chemical and Biological Engineering, The University of Sheffield, Mappin Street, Sheffield S1 3JD, United Kingdom

**Keywords:** iTRAQ, Quantitative proteomics, *Chlamydomonas reinhardtii*, Biofuels, Lipid production, Microalgae

## Abstract

Nitrogen stress is a common strategy employed to stimulate lipid accumulation in microalgae, a biofuel feedstock of topical interest. Although widely investigated, the underlying mechanism of this strategy is still poorly understood. We examined the proteome response of lipid accumulation in the model diatom, *Phaeodactylum tricornutum* (CCAP 1055/1), at an earlier stage of exposure to selective nitrogen exclusion than previously investigated, and at a time point when changes would reflect lipid accumulation more than carbohydrate accumulation. In total 1043 proteins were confidently identified (≥ 2 unique peptides) with 645 significant (*p* < 0.05) changes observed, in the LC-MS/MS based iTRAQ investigation. Analysis of significant changes in KEGG pathways and individual proteins showed that under nitrogen starvation *P. tricornutum* reorganizes its proteome in favour of nitrogen scavenging and reduced lipid degradation whilst rearranging the central energy metabolism that deprioritizes photosynthetic pathways. By doing this, this species appears to increase nitrogen availability inside the cell and limit its use to the pathways where it is needed most. Compared to previously published proteomic analysis of nitrogen starvation in *Chlamydomonas reinhardtii*, central energy metabolism and photosynthesis appear to be affected more in the diatom, whilst the green algae appears to invest its energy in reorganizing respiration and the cellular organization pathways.

## 1 Introduction

In the last few decades there has been a growing interest in developing microalgae as the third generation biofuel feedstock [1], [2], [3]. However, in order to develop economically viable processes for biofuel production using microalgae, a greater understanding of microalgal metabolism and its organization in effecting the accumulation of biofuel precursors (lipids and carbohydrates) is necessary. One of the most widely employed strategies that triggers the storage of energy reserves in microalgae is nitrogen limitation or depletion in the growth medium, which has been described for several species [4]. Recent studies have attempted to further understand this stress at the ‘-omic’ level, primarily using the model algal species *Chlamydomonas reinhardtii* [5], [6], [7], [8], [9]. However, given the diverse lineage of organisms classified under ‘microalgae’ [10], [11], such investigations are required in other lineages to develop a broader understanding of biofuel precursor synthesis and accumulation.

Diatoms play a significant role in the global carbon cycle, accounting for ~ 20% of total photosynthesis [12], and are of ecological significance. In addition, diatoms are also very interesting for conducting studies in algal physiology and applied phycology. Specifically, the marine diatom *Phaeodactylum tricornutum* has been used for aquaculture [13] and as a model for cell morphological investigations [14]. The marine nature of this organism is also of interest as a biofuel crop, as it allows for surmounting the water resource limitations associated with fresh water cultivations [15]. In this sense, *P. tricornutum* has been recommended as a favorable species for biodiesel production, with high lipid content (up to 61%) and lipid productivity (up to 26.75 mg L^− 1^ d ^− 1^) being reported [16], as well as having suitable lipid profiles for the derivation of biodiesel with desirable octane rating, iodine number and cloud point. The fact that its genome is sequenced [17], with descriptive information available in UniProt and KEGG, makes this species an excellent model organism for studying diatom based biofuel production [18]. As with many other microalgal species, *P. tricornutum* has been shown to increase lipid content in response to nitrogen stress [4]. Therefore, it is an excellent candidate to investigate the metabolic effect of the nitrogen trigger in diatoms, allowing its comparison with previous investigations from other taxonomic groups, such as Chlorophyta, and enabling a broader understanding of lipid accumulation in microalgae under this condition.

The effect of nitrogen stress has been examined previously at the molecular level in *P. tricornutum*, but this has been predominantly at the transcriptomic level using microarrays [19] and RNAseq [20], [21], [22]. In these investigations, changes in the proteome has been inferred from transcript expression profiles. Such approaches only provide assessment of the transcriptional control, disregarding the fact that both translational and degradation controls also affect the amount of protein present inside the cell [23]. This is of particular relevance in a nitrogen stress environment where protein degradation, as a way of nitrogen recovery, may play a significant role in fulfilling cellular nitrogen demands [6]. Hence, transcriptomic studies themselves cannot be relied upon to represent the true protein cellular levels [24], [25], [26]. These should either be supported with targeted protein analysis, such as western blots or multiple reaction monitoring, or a global proteomic investigation.

Whilst several proteomic investigations have been published in the Chlorophyta [9], [27], [28], [29], [30], [31], [32], [33], [34], there is limited information in other phyla. Within diatoms, *Thalassiosira pseudonana* [26], [35], [36], [37], [38], [39], [40], [41] and *P. tricornutum* [22], [37], [42], [43], [44], [45] have been the species most investigated. Some of these studies have referred to nitrogen stress in some form [22], [38], [39], [42], [44], [45]. Among these studies, the recent investigation by Ge et al. [42] reported proteomic changes using isobaric tags for relative and absolute quantitation (iTRAQ). iTRAQ utilizes amine linking isobaric tags to allow quantitative comparison of numerous proteins in an unbiased way and has become a popular tool for proteomics, being a major improvement compared to 2D SDS PAGE gel methodology [46], [47], [48]. Proteins detected in the work by Ge et al. [42] showed an increase in the carbohydrate metabolic processes (glycolysis and tricarboxylic acid cycle) and branched-chain amino acid catabolism, and a decrease in enzymes involved in cellular amino acid biosynthesis and photosynthesis. However, the proteomic analysis was done when *P. tricornutum* growth was well advanced and lipid accumulation was triggered by the natural depletion of nitrogen in the medium after 60 h of growth. In that kind of setting, the physiological state of *P. tricornutum* would be the result of the simultaneous change of other components in the medium in addition to the nitrogen concentration, and therefore, the observed proteomic changes could not be solely attributed to the nitrogen limitation. In the present analysis we aimed to study the effect of nitrogen starvation as the sole trigger of lipid accumulation in *P. tricornutum* by controlled removal of this key element from the culture medium, and by observing the changes relatively earlier than other investigations so far. The dynamics of *P. tricornutum* proteome reorganization were analysed using the iTRAQ methodology at 24 h after nitrogen removal, when lipid production in the nitrogen starved culture, compared to the nitrogen replete control conditions, was noticed to be at its highest, and when lipid accumulation appeared to take precedence over carbohydrate accumulation. The choice of the time point to analyse was based on the criteria to observe changes early enough under nitrogen depletion, but sufficiently delayed so as to differentiate the changes attributable to carbohydrate accumulation. We believe this aspect has not been addressed in previous investigations on the subject and would offer a more informed access to the relevant metabolic changes. Through the use of this mass spectrometry based proteomic quantification method and the unique design mentioned above, we aimed to increase current understanding of the relationship between nitrogen stress and lipid accumulation within microalgae. The results are also compared with previous analysis in *C. reinhardtii* [6], to gain insights into metabolic differences and similarities between different taxonomic affiliations. The ultimate goal is to acquire a better knowledge of the universality of the molecular mechanisms underlying the induction of lipid accumulation in microalgae that will lead to improved strategies for biofuel production from microalgae.

## 2 Materials and methods

### 2.1 Organism of study and medium

*P. tricornutum* (CCAP 1055/1) was obtained from the Culture Collection of Algae and Protozoa (CCAP, Oban, U.K.). F/2 + Si medium was prepared as described by CCAP diluting in seawater made with 33.6 g Ultramarine synthetic salts (Waterlife Research Industries Ltd. Middlesex, U.K.) per liter. Cultures were grown in either F/2 + Si medium (Nitrogen replete treatment) or F/2 + Si medium omitting sodium nitrate (Nitrogen deplete treatment).

### 2.2 Experimental approach

*P. tricornutum* was cultured in 250 mL bubble columns (40 mm diameter) sparged with air at 2.4 L min^− 1^. Filtered (0.22 μm) air was first passed through sterile water for humidification, before being introduced by silicone tubing to the bottom of the column providing both mixing and gas transfer. The top of the bubble column was sealed using a foam bung. The columns were placed in a water bath maintained at 25 °C and under 24 h continuous lighting with two side facing halogen lamps (230 V 11 W bulbs (OSRAM, Munich, Germany)). The lamps were placed horizontally across a series of columns. This arrangement resulted in an average light intensity of 200 μE m^− 2^ s^− 1^ for each column that varied by ± 50 μE m^− 2^ s^− 1^ along the length of the column, as measured using a Quantum Scalar Laboratory Radiometer (Biospherical Instruments, San Diego, CA, USA) in a water filled column. All columns in the experimental set-up received similar light exposure along the lines indicated above, such that the average for each column fell within sufficient to saturating light intensity for *P. tricornutum* [22], [49]).

Given that a considerable culture volume was required for proteomics, two batch cultures were carried out for each condition in triplicate. The first batch was used to generate sufficient biomass for profiling chlorophyll a, carbohydrate and (neutral) lipid profiles (collectively referred to as biochemical analyses, hereafter), and the second batch was used to generate the biomass for proteomics. The biochemical analyses were also carried out on a single time point from the second batch to ensure comparability of the batches. For both batches, the cultures were grown in nitrogen replete medium for 48 h reaching an optical density (OD_750nm_) > 0.4. Culture from four columns was then pooled and the combined optical density used to calculate the culture volume required for giving an OD_750nm_ of 0.2 upon re-suspension to 250 mL. The calculated culture volume was then harvested from the pooled culture by centrifugation at 3000 *g* for 5 min and resuspended in F/2 + Si medium with or without nitrate to generate the nitrogen replete and deplete treatments, respectively. These treatments were then sampled at 0, 6, 12, 18, 24, 36, 48, and 72 h, post resuspension, in the first batch to generate the biochemical profiles. Similarly sampling was done at 24 h for proteomic analysis and 72 h for biochemical analyses, with the second batch.

### 2.3 Biochemical analysis

For the first three time points the sample volume was 20 mL, whilst it was 15 mL for the subsequent five time points, for each biological replicate, for each treatment. Culture samples were pelleted in a pre-weighed 1.5 mL eppendorf tube by centrifugation at 3000 *g* for 5 min. Pellets were frozen before freeze drying for > 12 h in a Modulyo freeze drier (Edwards, Crawley, U.K.). Dried samples were weighed to determine the dry cell weight (DCW) and stored at − 20 °C. Chlorophyll a, carbohydrate and lipid analysis were conducted in the stored samples using modified versions of the Wellburn [50], Anthrone [51] and Nile red [52] methods respectively, as described in Longworth et al. [6]. In the same manner, chlorophyll *a*, carbohydrate and lipid analysis were conducted on the single time point sample collected from the second batch. There were thus four replicate data sets that were combined for the data analysis.

### 2.4 Microscopy

Samples for microscopy were taken from the proteomic experimental set (second batch) at 24 h post resuspension in each treatment condition by centrifugation of 1 mL sample at 3000 g for 5 min. After removing 950 μL, the pellet was resuspended in the remaining 50 μL and 10 μL was then placed on a glass slide with a cover slip on top. Visualization was done on an Olympus BX51 microscope (Olympus, Southend-on-Sea, U.K.) and images captured by using ProgRes CapturePro 2.6 (PandA, Berkshire, U.K.).

### 2.5 Proteomic sampling and processing

At 24 h after starting the treatments, two 50 mL aliquots were taken in each biological replicate and centrifuged at 3000 *g* for 10 min at 4 °C, then resuspended in 1 mL 500 mM triethylammonium bicarbonate buffer (TEAB) (pH 8.5) and transferred to protein low bind tubes. Samples were then stored at − 20 °C till all harvests were completed. Protein extraction was achieved by liquid nitrogen grinding. Stored cell samples were resuspended with 500 μL 500 mM TEAB (pH 8.5). Samples were immersed in a cooled sonication water bath for 5 min and subsequently ground using a mortar and pestle cooled by liquid nitrogen. Samples were then collected into a fresh protein low bind tube (Eppendorf, U.K.) and then immersed in a cooled sonication water bath for a further 5 min and sonicated for two cycles with a Micro tip Branson sonifier (Enerson, Danbury, CT, USA). Subsequently, samples were centrifuged at 18,000 *g* for 30 min at 4 °C to separate the soluble and insoluble fractions. After quantifying using RCDC (BioRad, U.S.A), 100 μg of protein was acetone-precipitated before being resuspended in 30 μL 500 mM TEAB (pH 8.5) with 0.1% sodium dodecyl sulphate. Proteomic samples were then reduced, alkylated, digested and labelled with the 8-plex iTRAQ reagents (AB Sciex, Framingham, MA, USA), as described in the manufacturer's protocol. To assess the proteomic changes occurring within *P. tricornutum* under nitrogen stress an 8-plex iTRAQ experiment was designed. iTRAQ labels 114, 113 and 119 were used for nitrogen replete biological triplicate cultures and 116, 117 and 118 for nitrogen depleted biological triplicate ones. Note that labels 115 and 121 were intended for analysing the samples from a silicon stress experiment. However, silicon was not effectively depleted and thus these proteomic results were incorporated into the nitrogen replete ones.

### 2.6 Off line HPLC fractionation and clean-up

High-resolution hydrophilic interaction chromatography (HILIC) was carried out using an Agilent 100-series HPLC (Agilent, Wokingham, UK). One iTRAQ labelled sample was resuspended in 100 μL buffer A (10 mM ammonium formate, 90% ACN, pH 3 (adjusted with formic acid (FA)). The resuspended sample was loaded onto PolyHydroxyethyl A column, 5 μm particle size, 20 cm length, 2.1 mm diameter, 200 Å pore size (PolyLC, Columbia, MD, USA). With a flow of 0.5 mL min^− 1^ buffer A was exchanged with buffer B (10 mM ammonium formate, 10% ACN, pH 4 (adjusted with (FA)) to form a linear gradient as follows: 0% B (0–5 min), 0–15% B (5–7 min), 15% B (7–10 min), 15–60% B (10–50 min), 60–100% B (50–55 min), 100% B (55–65 min), 0% B (65–75 min). Fractions were collected every minute from 18 min through to 41 min followed by three, 3 min fractions to 50 min. The fractions were vacuum centrifuged, before being cleaned up using C18 UltraMicroSpin Columns (Nest, Southborough, MA, USA) according to the manufacturer's guidelines.

### 2.7 LC-MS/MS

RPLC-MS was conducted using an Ultimate 3000 HPLC (Dionex, Sunnyvale, CA, USA) coupled to a QStar XL Hybrid ESI Quadrupole time-of-flight tandem mass spectrometer (Applied Biosystems (now ABSciex), Framingham, MA, USA). Samples were resuspended in 20 μL buffer A (3% ACN, 0.1% FA) before loading 9 μL onto a Acclaim PepMap 100 C18 column, 3 μm particle size, 15 cm length, 75 μm diameter, 100 Å pore size (Dionex, Sunnyvale, CA, USA). With a flow of 300 μ min^− 1^, buffer A was exchanged with buffer B (97% ACN, 0.1% FA) to form a linear gradient as follows: 3% B (0–5 min), 3–35% B (5–95 min), 35–90% B (95–97 min), 90% B (97–102 min), 3% B (102–130 min). The mass detector range was set to 350–1800 *m*/*z* and operated in the positive ion mode saving data in centroid mode. Peptides with + 2, + 3, and + 4 were selected for fragmentation. The remaining sample was subsequently injected in the same manner to acquire two RPLC-MS runs for each submitted fraction.

### 2.8 Data analysis

Proteomic identifications were conducted using Mascot, Ommsa, X!Tandem, Phenyx, Peaks and ProteinPilot for searching against the Uniprot reference proteome for *Phaeodactylum tricornutum* (Uniprot id 10,465). Each search was conducted with a decoy database formed using reversed sequences (Mascot, Ommsa, X!Tandem and ProteinPilot) or randomized sequence (Phenyx and Peaks). Searches were restricted to a peptide false discovery rate (FDR) of 3% prior to decoy hits being removed and peptide spectral matches from the six search engines being merged using an R based script that was also used to remove those showing disagreement in terms of peptide assignment or protein identification between the search engines. Where protein groups were clustered, such as with Mascot, the most common identification between the search engines was selected. Separately, for quantification, the reporter ion intensities for each peptide spectral match (PSM) were extracted and matched to the merged results. Thus only reporter ion intensities from PSM's matched by the above merging contributed to the protein reporter ion intensities, each PSM match having equal weighting whether identified by single or multiple search engines. Variance stabilization normalization, isotopic correction and median correction were performed on the label intensities before averaging by protein and performing a *t*-test between replicate conditions to determine significance and fold change. (Supporting Information Fig. S1).

KEGG analysis was derived using the KEGG “Search&Color Pathway” tool [53]. Proteins with a significant (*p*-value < 0.05) positive fold change were labelled with “blue” whilst proteins with a significant (*p*-value < 0.05) negative fold “red”.

Gene ontology (GO) annotations were identified using the functional annotation tool DAVID [54], [55]. The GO terms were then grouped into biological concepts as shown in Supporting Information Table S1. To determine the relative change, the number of proteins identified as increasing within a class was divided by the number of proteins identified as decreasing with the change being log transformed (base 10). This provides an observation of the relative change observed in each species balanced on 0 for each grouping of GO terms.

## 3 Results and discussion

### 3.1 Biochemical characterization under nitrogen stress

**Fig. 1:**
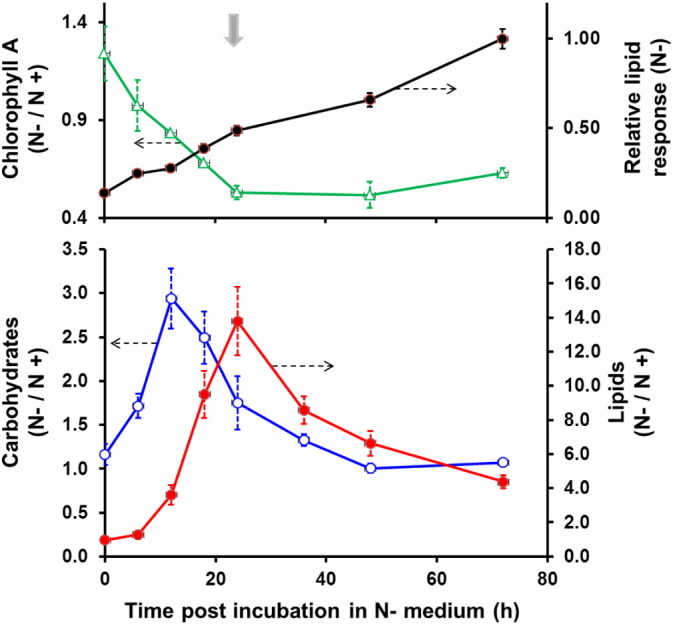
Ratio of biomass normalized biochemical responses under nitrogen deplete (N −) compared to nitrogen replete (N +) condition; lipids by Nile red fluorescence (lower panel), carbohydrates (lower panel); and chlorophyll A (upper panel). The lipid response in N − condition (upper panel) is the Nile-red fluorescence response that is normalized to the maximum observed for the condition. Error bars refer to standard error about the mean of the four biological replicates. The block arrow at 24 h, in the upper panel, indicates the sampling point for proteomics. (For interpretation of the references to colour in this figure legend, the reader is referred to the web version of this article.)

**Fig. 2:**
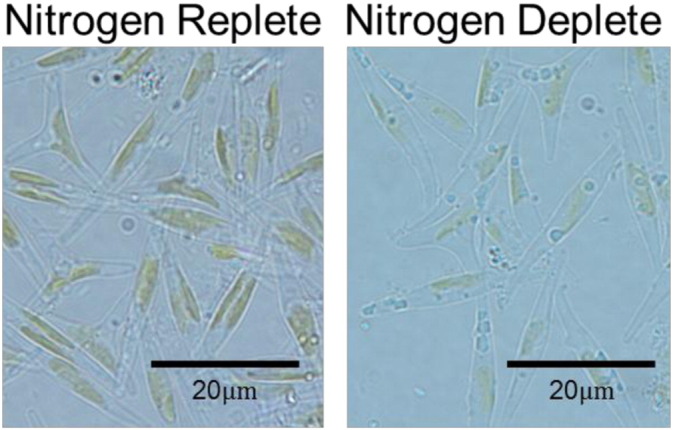
Microscope images at 100 × magnification of *P. tricornutum* 24 h after transfer to test conditions.

The assessment of *P. tricornutum* biochemical changes under the exclusive influence of nitrogen deprivation is shown in Fig. 1. Here, the ratio of the relative biomass normalized response of the variables under nitrogen depletion with respect to the control (nitrogen replete scenario) can be studied. As can be seen from the plot, both carbohydrates and lipids are produced at higher levels under nitrogen depleted conditions compared to the replete scenario, in the initial stages of the exposure. The carbohydrate levels peak initially (at 12 h post incubation) reaching a maximum of 3 fold increase under nitrogen depleted condition. Neutral lipid levels are significantly higher in relative terms at all times, and peak latter than carbohydrates, at 24 h. This initial increase in carbohydrates followed by increase in lipids is as was observed in *C. reinhardtii* under nitrogen stress [6]. As can be seen from the upper panel of the figure, the ratio of Chlorophyll A response decreased rapidly over the first 24 h. This was confirmed by the visible decrease in chloroplast content in the nitrogen depleted treatment as observed under the microscope (Fig. 2).

Considering the results observed in Fig. 1 and in order to investigate changes in the proteome associated with the lipid accumulation, a sampling point of 24 h post resuspension in nitrogen free medium was chosen for conducting the proteomics analysis. The chosen time point is one where the lipids were being accumulated at a rate higher than in the control condition, but one where the relative carbohydrate accumulations were minimal, suggesting a switch in resources from carbohydrate accumulation to lipid accumulation. A snapshot of metabolism at this time point can be considered to reflect changes that are more relevant to lipid accumulation than those attributable to carbohydrate accumulation.

### 3.2 Biochemical analysis of proteomic culture setting

To ensure culture comparability to the biochemical profile data set, samples for biochemical and microscopy analysis were also taken along with those for proteomic analysis at 24 h post resuspension. A *t*-test showed a statistically significant (*p*-value < 0.05) increase in carbohydrates and lipids when cultures were under nitrogen stress for 24 h. Conversely, pigmentation showed a significant reduction in the nitrogen depleted treatment (Supporting Information Fig. S2). Concurrent with proteomics and biochemical analysis, 1 mL of culture was also prepared for microscopy (Fig. 2). The nitrogen stressed cells were observed to have reduced pigmentation, which is in accordance with the observations made for the Chlorophyll *a* and Carotenoids concentration (Supporting Information Fig. S2).

### 3.3 Overview of proteomic data

**Fig. 3:**
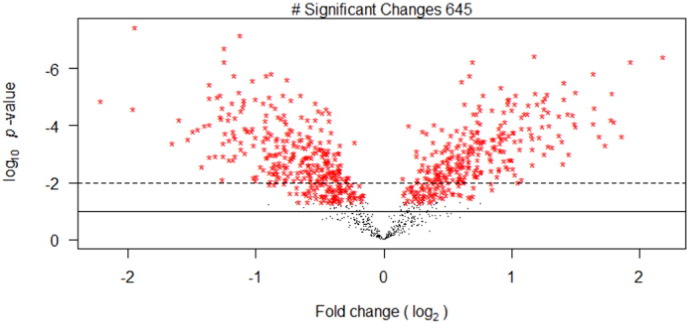
Volcano plot of proteins identified showing fold change and statistical significance of change. Significant changes to a *p*-value < 0.05 are indicated by red *. The *p*-value cut-off of 0.01 and 0.1 are indicated by a dotted and solid line respectively. (For interpretation of the references to colour in this figure legend, the reader is referred to the web version of this article.)

Within the proteome dataset, 23,544 spectra were matched to peptide and protein without disagreement among the six search engines, each of which were limited to a false discovery rate of 3% at the peptide level. The derived PSM list represented 7777 unique sequences matched to 1761 proteins of which 1043 had two or more unique peptides (Supporting Information Table S2). To assess sample arrangement, hierarchal clustering and principal component analysis (Supporting Information Fig. S3) was performed on the merged PSM list. From this analysis, it can be seen that the nitrogen stress replicates cluster apart from the replete cultures and is responsible for > 80% of the variation between the samples. The list of PSM(s) was then processed to provide the degree and significance of the change between the two treatments (Supporting Information Table S3). Between the nitrogen replete and deplete conditions, 645 significant changes (Fig. 3) were observed (*p* ≤ 0.05), which corresponds to 62% of the confidently identified proteins. Though double that observed by Ge et al. (29% [42]) this high level of statistically significant change is comparable with other studies of nitrogen stress in algae (53% [6] and 33% [9] for *C. reinhardtii*, 57% [27] for *Chlorella vulgaris*). For biological description two sets of statistically significant proteins were used. The 645 changes identified as showing a significant difference (*p* ≤ 0.05) were used for pathway and gene ontology analysis, which requires deduction of hypotheses based on protein clusters rather than individual observations. A more stringent significance level (*p* ≤ 0.01) comprising of 498 differences was used for direct hypothesis derivation in Table 1.

### 3.4 Resourcing of internal nitrogen, scavenging and the reduction of lipid degradation

**Fig. 4:**
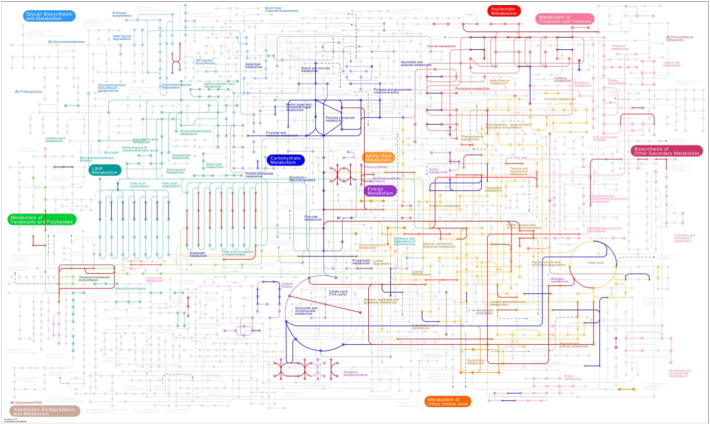
Metabolic pathway diagram from KEGG. showing proteins with significant (*p*-value < 0.05) increase or decrease in abundance in blue and red respectively. (For interpretation of the references to colour in this figure legend, the reader is referred to the web version of this article.)

Significant changes (*p* < 0.05) between nitrogen replete and deplete conditions were used to colour KEGG maps. The overall map of the metabolism is shown in Fig. 4 (specific pathway maps grouped by concept are shown in Supplementary Information Figs. S5–10). Given limited annotation of KEGG available for *P. tricornutum*, the most significant changes were further investigated individually. Within the dataset, the abundance of 498 confidently identified proteins (≥ 2 unique peptides) was significantly (*p* < 0.01) altered. These were matched to protein names using UniProt. Discounting those described as ‘Predicted Protein’ or ‘Predicted protein (Fragment)’ 193 identifications with descriptive names were grouped using the protein name and information provided on the UniProt entry page (Table 1). Both KEGG and individual analysis showed significant trends in the reorganization of *P. tricornutum* proteome under nitrogen stress, mostly towards maximizing the use of the remaining nitrogen. Among others, those pathways involved in increasing the availability of the intracellular nitrogen and minimising its loss were favoured.

Amino acid synthesis was reorganized between the different families, as is suggested by the decrease in the synthesis of the families of the aromatic-like, aspartate-like and pyruvate-like amino acids (Supporting Information Fig. S6 and Table 1). There was, however, observation of an increase in serine tRNA, suggesting that whilst decreased in general, proteins associated with some amino acid synthesis may have increased. In contrast to previous reports that suggest a general decrease of amino acid synthesis in *P. tricornutum* [45], grouping the amino acid production based on their type (e.g. aromatic and hydrophobicity) did not reveal any meaningful trend. The ample coverage of the decrease of ribosomal proteins (Supporting Information Fig. S5 and Table 1) confirmed the reduction of protein synthesis associated with nitrogen stress that has been reported previously [34], [56]. This would be linked to the cellular need to economize the use of the available nitrogen. Given the nature of the stress condition, it was also expected that nitrogen scavenging would be strongly promoted within the cell as a way of supplying nitrogen demands. In this sense, focusing on the nitrogen metabolism pathway, proteins with greater abundance in the nitrogen depleted treatment included aliphatic amidase and formidase, both of which are known to free ammonia from other macromolecular compounds (Table 1) [57]. Conversely, nitrate reductase, responsible for converting the available nitrate in the medium to nitrite in the initial step of nitrate assimilation, was decreased, contrasting with recent studies in *P. tricornutum* [22], likely due to the fact that in these studies the effect of nitrogen limitation rather than nitrogen starvation was addressed. Similar down-regulation has been reported for the diatom *T. pseudonana* under nitrogen starvation and iron stress [38], [58] that also coincided with an increase of the enzyme urease in the former, matching the increased abundance of the urea transporter found in this study. The possession of a complete urea cycle by the diatoms has been suggested to be a way of increasing the efficiency of nitrogen re-assimilation from catabolic processes [22], [59]. An increased abundance of the proteins involved has been reported to be linked to the increase in the glycolytic pathway of *P. tricornutum* facing nitrogen deprivation [60]. In conclusion, this increase in nitrogen scavenging when seen with the reduction in the nitrogen assimilation enzyme suggests a more active rather than a passive response to the nitrogen stress focused on intracellular nitrogen recycling.

The possession of this active nitrogen scavenging strategy might also be demonstrated by the increases in proteasome proteins and the changes of endocytosis and phagosome. KEGG analysis showed an increase in ‘Endocytosis’ and ‘Phagosome’ activity under nitrogen stress (Supporting Information Fig. S10). Such increases in phagosomal activity have previously been reported for other algae under nitrogen stress, for example in Bihan et al.'s proteomic study on *Ostreococcus tauri* [61], This would suggest a scavenging response of microalgae under nitrogen deprivation. In this sense, when facing reduced nitrogen availability, *P. tricornutum* cells might enhance the intake and processing of extracellular debris and perhaps attempts to consume other organisms such as bacteria to obtain additional nitrogen supplies. Thus, nitrogen stress could be suggested to induce phagotrophy [62], [63], In addition to external nitrogen retrieval, many of the proteins associated with endocytosis and phagocytosis have been reported to be similarly involved in autophagy [64], [65]. Transcriptional evidence of a link between nitrogen stress and autophagy induction has been previously shown in the chlorophyta *Neochloris* [66].

Pathways associated with fatty acid metabolism were also significantly changed under nitrogen stress, coinciding with the previously described enhancement in the lipid content (Supporting Information Fig. S9 and Table 1). Increases in KEGG pathways included ‘biosynthesis of unsaturated fatty acids’, ‘fatty acid biosynthesis’ and “short chain fatty acids”; and a relative decrease was observed in ‘fatty acid elongation’ and ‘fatty acid metabolism’. Coinciding with previous reports [34], [42], individual protein changes also displayed an active dynamism of the proteome involved in this metabolic pathway, implying an increased abundance of enzymes key to lipid biosynthesis, such as acyl-carrier proteins and malonyl-CoA:ACP transacyclase. Additionally, a decrease in fatty acid catabolism related proteins was found, suggesting that a down-regulation in the degradation of fatty acids might be a key metabolic route for explaining lipid accumulation under nitrogen stress conditions. These results have been shown previously [6], [66], [67] and are supported by recent reports of the preservation of existing triacylglyderides after nitrogen stress situations [68]. Similar dynamism of the proteins related to the fatty acid synthesis and degradation has been reported previously for Chlorophyta [6], [28], [66]. These results contradict those shown by the transcriptomic study conducted in *P. tricornutum* by Valenzuela et al. [21], highlighting the inappropriateness of using transcriptomic data to infer proteomic changes, as has been previously reported [24], [25], [26]. The discord between these findings might suggest a translational control for proteins associated with fatty acid biosynthesis and degradation that would not be necessarily reflected at the transcriptomic level.

### 3.5 Preference of the central energy metabolism over photosynthetic pathways

The photosynthetic pathway was significantly down-regulated under nitrogen stress in *P. tricornutum*, as observed by a decrease in the relative abundance of the most important enzyme in the carbon fixation pathway (RuBISCO), and the general decreased abundance of key proteins of photosynthesis such as the light harvesting proteins and the photosynthetic electron transport system (e.g., fucoxanthin chlorophyll *a*/c, ATP synthase, PSI, PSII and cytochrome c, Table 1). This observation matches a similar trend detected by the KEGG analysis (Supporting information S8) and the decrease in pigment content described previously (Fig. 1). Further, it is in agreement with previous studies both in *P. tricornutum* and other algae, supporting ample evidence on the close linkage between carbon and nitrogen metabolism [6], [9], [38], [45]. Such degradation of the photosynthetic pathway would be due to the fact that photosynthetic proteins (including pigments such as chlorophyll a) have a high content of nitrogen, and therefore, under conditions of nitrogen scarcity, cells tend to actively down-regulate their synthesis in order to preserve the little nitrogen that is left and to divert it to the synthesis of those proteins that are essential for cell maintenance [6], [56].

The reorganization of the proteome under nitrogen starvation would also have an impact on the central energy metabolism. Acetyl CoA plays an important role in the carbon partitioning for oil accumulation within the cell, and therefore, metabolic pathways would be redirected to increase of the availability of this metabolite in the cell. In addition, fatty acid synthesis requires high levels of ATP and NADPH that would be generated through a switch from a gluconeogenic to a glycolytic metabolism. In this sense, in our study an increased abundance of those proteins involved in the Kreb's cycle, the glycolysis and the oxidative pentose phosphate pathways were observed. Conversely, those enzymes regulating the glycolytic and the gluconeogenic pathways reported decreased abundance(Table 1 and Supporting information S7), confirming previous reports for diatoms and cyanobacteria under nitrogen stress [20], [22], [38], [42], [45], [69].

Finally, nine proteins with antioxidant properties were increased under nitrogen stress, suggesting a change in the concentration of reactive oxygen species (ROS) within the cellular environment (Table 1). An increase in ROS has been reported to be a major source of cellular damage under abiotic and biotic stresses in plants [70]. Specifically, ROS increases under nitrogen starvation conditions are closely linked with the malfunctioning of the photosynthetic pathway. Nitrogen uptake and metabolism require reducing equivalent power and ATP that under nitrogen deprived conditions tend to accumulate, causing metabolic imbalance and leading to the generation of oxidative stress. Nitrogen is also required for the synthesis of photosynthetic proteins, especially light harvesting proteins, and, as has been explained before, its lack tends to slow-down the electron flow through the photosynthetic apparatus, in turn causing the production of more ROS. Therefore, it can be hypothesized that the observed increase in antioxidant proteins is a mechanism used by *P. tricornutum* to limit this oxidative stress damage, as has been reported for algae facing other or similar stressful conditions [38], [45], [71]. Another indication of the stress to which *P. tricornutum* was subjected to under nitrogen starvation is the increased abundance of the heat-shock protein HSP20. Heat shock protein expression has been reported to be triggered in microalgae growing under stressful conditions [71], including nitrogen stresses [45]. However, it is also interesting that, while HSP20 was increased, other heat shock proteins, which have also been described to be present in stress responses, showed an opposite pattern, suggesting their possible differential role in the cell.

### 3.6 Comparison of the response of *P. tricornutum* and *C. reinhardtii*

**Fig. 5:**
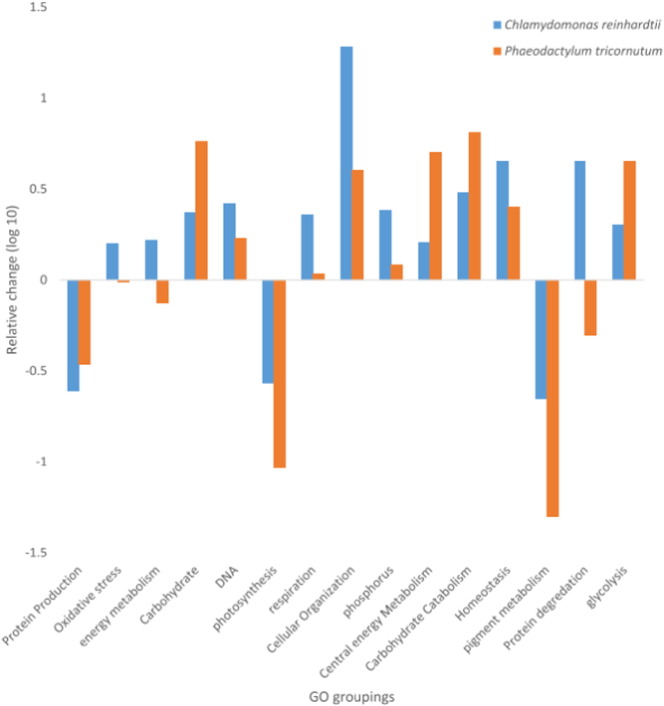
Comparison of proteomic response in *P. tricornutum* and *C. reinhardtii.* The relative change within each GO grouping between the number of proteins assigned with increased and decreased abundances are shown.

To investigate differences in the proteome response under nitrogen stress between very different microalgae taxonomic affiliations such as *Bacillariophyceae* and *Chlorophyceae*, the results obtained in this study for *P. tricornutum* and the published earlier work of ours for *C. reinhardtii* [6] were compared. Although there were differences in terms of sampling time points and culture conditions between the studies, both were conducted under active increase of cellular lipid content and thus this comparison is of interest. As far as we know this is the first study aiming at such comparison under situations of nitrogen starvation. Observing the changes in the GO groupings did not show any strong unidirectional change between the two species, however observation of the relative changes in proteins captured showed some differences (Fig. 5).

The direction of the protein abundance change in the number of proteins was the same for both species with two exceptions, those proteins that are involved in energy metabolism and protein degradation. Both showed increases in *C. reinhardtii* and decreases in *P. tricornutum. P. tricornutum* also demonstrated more consistent protein abundance changes involved in photosynthesis, pigment metabolism, carbohydrates metabolism, central energy metabolism and glycolysis than *C. reinhardtii*; suggesting that the reorganization of the proteome in this species towards these metabolic pathways was more important.

Of special note is the markedly larger number of proteins involved in the photosynthetic pathway that were reduced in abundance in *P. tricornutum.* This might be due to the differences in the photosynthetic machinery between both species in terms of energy dissipation pathways and photosynthetic components of the electron transport system. Accessory pigments are very important in diatoms for dissipating excess energy due to the photosynthetic activity and, given their high nitrogen content, tend to be scavenged very early in the onset of nitrogen starvation [56]. The larger number of proteins with increased abundance in central energy metabolism, mainly the GO terms acetyl-CoA and acyl-CoA metabolic processes (Supporting Information Table S1), and glycolysis in *P. tricornutum* also suggest the relevance of these pathways in the cellular response to nitrogen starvation. These are likely involved in increasing the availability of the acetyl-CoA, chemical energy and reductant power required for lipid biosynthesis (see more details above). The relative higher increase of glycolysis and carbohydrate catabolism also might indicate that *P. tricornutum* tends to mobilize carbon stores rather than increase them under nitrogen scarcity, as has been previously reported [38].

Conversely, *C. reinhardtii* had more proteins regulated that relate to cellular homeostasis, respiration, phosphorous metabolism, DNA metabolism and cell organization compared to *P. tricornutum*; of practical note are the relatively large number of proteins involved in respiration and cellular organization. In our previous work [6] *C. reinhardtii* was grown in the presence of organic carbon and the observed higher number of respiratory proteins could be explained by the diversion of the metabolism towards heterotrophy as a consequence of the compromise of the photosynthetic pathway in conditions of nitrogen scarcity. This switch from photoheterotrophic to heterotrophic metabolism has been described before for this species under conditions of Iron deprivation [72]. The respiratory pathway would be used for generating chemical energy and reductant power needed for lipid biosynthesis. Induction of gametogenesis in *C. reinhardtii* under nitrogen stress has been reported [73], and the active increased abundance of cellular organization proteins (mainly cytoskeletal proteins - personal comment by the authors) observed here might play an important role in such physiological response.

Finally, *C. reinhardtii* seemed to be more susceptible than *P. tricornutum* to the oxidative stress caused by nitrogen starvation, as suggested by the observed relatively higher number of oxidative stress proteins. Oxidative stress increase in microalgae under nitrogen starvation conditions has been described widely in the past [38], [45], [70], [71], and has been related to the damage of the photosynthetic electron system proteins due to the nitrogen scarcity. However, the results of our comparison would suggest that there would be differences in both species in the way they counteract the oxidative stress damage, with a higher protein response in *C. reinhardtii* that might be associated to a different source of oxidative stress. While *P. tricornutum* remained photoautotrophic when growing under nitrogen starvation and therefore mostly the oxidative stress was caused by an inefficient functioning of the photosynthetic pathway and the xanthophyll cycle, *C. reinhardtii* growth conditions were mixotrophic (acetate as a source of organic Carbon) and in conditions of Nitrogen starvation would switch towards a heterotrophic growth and the oxidative stress associated to the increase in respiration would be added to that caused by the damaged photosynthetic pathway.

It must be noted that the above comparison is not comprehensive, taking into consideration all the relevant physiological and biological differences between the organisms and cultivation conditions. Nevertheless, it provides vital clues that will enable us to explore and develop a better understanding of microalgal metabolism needed for developing viable strategies for bioenergy generation.

## 4 Conclusions

In the present study, the biochemical and proteomic changes associated with nitrogen starvation as a trigger for enhancing lipid production was addressed in *P. tricornutum* and compared with those previously described for *C. reinhardtii*. From biochemical analysis, it can be concluded that nitrogen stress increases energy storage molecules in *P. tricornutum*. This increase would be coupled with a decrease in photosynthetic pigments. We examined the proteome at an earlier stage of exposure to exclusive nitrogen starvation than has been reported, but at a time point when changes attributable to lipid accumulation can be captured in preference to those due to carbohydrate accumulation. Through the use of an iTRAQ methodology, 1043 proteins were confidently identified, of which 645 were shown to be significantly altered abundance under nitrogen stress. This represents a 17-fold increase with respect to the number of proteins detected in previous nitrogen stress assessments of *P. tricornutum*, and as such provides greater understanding of the effects of nitrogen stress in this model diatom species.

The extent to which the proteome changes in response to nitrogen stress has been demonstrated to be > 60%, with over 60% of the confidently identified proteins being significantly changed (*p*-value < 0.05) in abundance. Several patterns of response have been identified within the proteome highlighting increased scavenging of nitrogen and the reduction of lipid degradation, as well as stimulation of central energy metabolism in preference to photosynthetic pathways.

The GO comparison of *P. tricornutum* and *C. reinhardtii* conducted here highlights important differences in the degree of protein investment among the different metabolic pathways. In this sense, under nitrogen starvation, whilst *P. tricornutum* might reorganize its proteome by largely decreasing the number of photosynthetic proteins and increasing the ones involved in central energy metabolism, *C. reinhardtii* appears to invest in cellular reorganization, respiration and oxidative stress response.

The following are the supplementary data related to this article.

Supplementary figures. Image 1

Table S1 Reference table for Gene ontology groupings. Table S1

Table S2 Peptide table of six merged search engines. Table S2

Table S3 Fold change and significance table. Table S3

## Conflict of interest

The authors declare no competing financial interest.

## Author contributions

JL contributed to the conception, design, data acquisition and drafting of the article, DW contributed in the data acquisition and drafting of the article, MHO contributed to the design, and drafting of the article with critical insights and input, PCW contributed to the design, conception and drafting of the article, and SV contributed to the conception, design, supervision and drafting of the article. All authors give their final approval of the submitted manuscript.

## Figures and Tables

**Table 1:** Table of all significant (*p* < 0.01) changes observed omitting “Predicted Proteins”. Each protein is reported with its Uniprot ID, Descriptive name, Number of unique peptides and fold change observed under nitrogen stress. Positive fold changes are shown in bold.

Uniprot ID	Protein name	# Peptides	Fold Change
	Hydrophilic amino acid synthesis		
B7GEJ6	Acetylornithine aminotransferase	7	1.28
B7G5H9	Aspartokinase	2	− 1.50
B7GBH2	Delta l-pyrroline-5-carboxylate synthetase	12	− 1.35
B7G3A2	Diaminopimelate decarboxylase	6	1.31
	Hydrophobic amino acid synthesis		
B7FUP6	2-Isopropylmalate synthase	6	− 1.55
B7FRJ9	3-Deoxy-7-phosphoheptulonate synthase	15	− 1.69
B7FT14	Adenosylhomocysteinasee	18	− 2.36
B7G2T9	Carboxy-lyase	3	− 1.27
B7FS76	Chorismate synthase	2	1.27
B7G117	O-acetylhomoserine	8	− 1.22
	Other amino acid synthesis		
B7FT50	Asparagine synthetasee	4	1.53
B7G5Z8	Glycine decarboxylase p-protein	16	− 1.63
B7FZB0	Synthase of glutamate synthase	18	1.33
	Photosynthesis		
A0T0C9	Apocytochrome f	17	− 1.91
A0T0D1	ATP synthase epsilon chain, chloroplastic	7	− 1.84
A0T0F1	ATP synthase subunit alpha, chloroplastic	40	− 2.18
A0T0E9	ATP synthase subunit b, chloroplastic	5	− 2.89
A0T0E8	ATP synthase subunit b’, chloroplastic	2	− 2.80
A0T0D2	ATP synthase subunit beta, chloroplastic	49	− 2.37
A0T0F0	ATP synthase subunit delta, chloroplastic	3	− 2.26
A0T0A3	Cytochrome b559 subunit alpha (PSII reaction center subunit V)	6	− 2.73
B7FSN7	Delta-aminolevulinic acid dehydratase	8	− 1.27
B7G3I6	Fucoxanthin chlorophyll a/c protein, deviant	9	− 1.71
Q41093	Fucoxanthin-chlorophyll a-c binding protein E, chloroplastic	11	− 1.65
A0T0B5	Magnesium-chelatase subunit I	16	− 2.42
B7FZ96	Oxygen-evolving enhancer protein 1	14	− 1.35
A0T0B9	Photosystem I ferredoxin-binding protein	28	− 1.48
A0T0M1	Photosystem I protein F	9	− 1.54
A0T0M6	Photosystem I reaction center subunit XI	4	− 1.78
A0T096	Photosystem II CP43 chlorophyll apoprotein	15	− 2.49
A0T0B2	Photosystem II CP47 chlorophyll apoprotein	17	− 3.15
A0T097	Photosystem II D2 protein	3	− 2.69
A0T0H5	Photosystem II reaction center psb28 protein	9	1.78
A0T0G9	Photosystem Q(B) protein	3	− 1.85
B7FZL9	Phytoene dehydrogenase	2	− 1.67
B7FRW2	Protein fucoxanthin chlorophyl a/c	24	− 1.53
B7FQE0	Protein fucoxanthin chlorophyl a/c	4	− 1.40
B7FQE1	Protein fucoxanthin chlorophyl a/c	7	− 1.63
B7FR60	Protein fucoxanthin chlorophyl a/c	4	1.87
B7FRW4	Protein fucoxanthin chlorophyl a/c	3	− 1.49
B7FV42	Protein fucoxanthin chlorophyl a/c	4	− 1.77
B7G6Y1	Protein fucoxanthin chlorophyl a/c	3	− 1.85
B7G955	Protein fucoxanthin chlorophyl a/c	3	− 1.72
B7GCV9	Protein fucoxanthin chlorophyl a/c	3	3.13
B5Y3F4	Protoporphyrin IX magnesium chelatase, subunit H	6	− 2.14
B7GDU9	Protoporphyrinogen oxidase	2	− 1.65
B7FUT6	Uroporphyrinogen decarboxylase	10	− 2.19
B7FUR6	Violaxanthin deepoxidase	4	1.56
	Carbon fixation		
Q9TK52	Ribulose bisphosphate carboxylase large chain	38	− 2.33
A0T0E2	Ribulose-1.5-bisphosphate carboxylase/oxygenase small subunit	5	− 1.92
	Energy metabolism		
B7FXB6	6-Phosphogluconate dehydrogenase, decarboxylating	6	1.66
B5Y3C9	Cytochrome b6-f complex iron-sulfur subunit	11	− 1.72
Q8GTB5	Cytochrome c6 (Precursor cytochrome *c*6)	4	1.79
B5Y578	Cytochrome c6, cytochrome *c*553	10	1.43
B7FRC1	Cytosolic aldolase	8	1.90
Q9M7R3	Cytosolic glyceraldehyde-3-phosphate dehydrogenase	18	1.89
Q84XB5	Fructose-1.6-bisphosphate aldolase	16	1.42
B7GDK9	Glucose-6-phosphate isomerase	7	1.50
B7G6T5	Glutamine-fructose-6-phosphate transaminase	4	1.61
B7G518	Isocitrate lyase	3	− 1.90
B7FYD8	Kinase adenylate kinase	3	− 1.53
B7G0K7	Ligase succinate-coa ligase	3	1.58
B7G9G3	Lipoamide dehydrogenase	15	1.19
B7FYT9	Malate synthase	6	− 1.41
B7GCG9	PFP pyrophosphate dependent phosphofructokinase	11	1.58
B7GEI2	Phosphoglycerate mutase	4	1.87
B7G492	Phosphomannose mutase	5	1.66
B7GEF2	Plastidic enolase	17	1.20
B7G0M9	Precursor of ATPase ATPase gamma subunit	11	− 1.58
B7FZE1	Precursor of dehydrogenase pyruvate dehydrogenase E1, alpha and beta subunits	24	1.62
F1SXA3	Putative phosphoenolpyruvate carboxykinase	5	1.54
B7FZG7	Pyruvate kinase	6	1.40
Q2TSW8	Pyruvate kinase	3	− 1.25
Q2TSW9	Pyruvate kinase	7	1.37
Q2TSX0	Pyruvate kinase	5	1.34
B5Y5N6	Succinate dehydrogenase flavoprotein	19	1.66
B7GA40	Succinate dehydrogenase iron sulfur protein	5	2.59
B7FUU0	Transketolase	36	− 1.37
B7G5R3	Transketolase	10	1.58
B7FT67	Triosephosphate isomerase	6	1.42
B7G3C1	Triosephosphate isomerase	4	1.46
	Fatty acid biosynthesis		
B7G1R8	3-oxoacyl-[acyl-carrier protein	11	1.71
B7GCM0	3-oxoacyl-[acyl-carrier-protein] synthase	10	1.51
B7G7H8	3R-hydroxyacyl-[acyl carrier protein] dehydrase	4	1.41
A0T0F8	Acyl carrier protein	2	1.96
B7FRX6	Acyl carrier protein	3	2.00
B7G3D4	Malonyl-CoA:ACP transacylase	2	1.41
Q2TSX2	Mitochondrial glyceraldehyde-3-phosphate dehydrogenase	2	1.53
E6Y9B3	Stearoyl-ACP desaturase	2	1.48
	Fatty acid catabolism		
B5Y4D9	Long chain acyl-CoA synthetase	2	− 1.78
B7FXX6	Long chain acyl-coa synthetase	7	− 1.31
B7FW77	Peroxisomal 2.4-dienoyl-CoA reductase	2	− 2.03
B5Y5R5	Short chain acyl-coenzyme A dehydrogenase	5	− 1.28
	Nucleotide biosynthesis		
B7FP55	Inosine-5′-monophosphate dehydrogenase	2	− 1.44
B7FPE8	Nucleoside diphosphate kinase 1	6	1.25
B7FR80	Nucleoside diphosphate kinase 3	6	1.55
	Translation		
A0T0J8	30S ribosomal protein S13, chloroplastic	5	− 1.45
A0T0B3	30S ribosomal protein S14, chloroplastic	2	− 2.04
B7FU91	30S ribosomal protein S15	3	− 1.82
Q5D704	30S ribosomal protein S16, chloroplastic	2	− 1.57
A0T0E0	30S ribosomal protein S2, chloroplastic	3	− 1.71
A0T0I5	30S ribosomal protein S3, chloroplastic	4	− 1.66
A0T0J5	30S ribosomal protein S5, chloroplastic	9	− 1.51
A0T0K5	30S ribosomal protein S7, chloroplastic	3	− 1.43
A0T0K2	30S ribosomal protein S9, chloroplastic	2	− 2.56
B7FPA1	40S ribosomal protein S12	7	− 1.66
B7FPM3	40S ribosomal protein S3a	8	− 1.87
B5Y4X4	40S ribosomal protein S6	15	− 2.28
B7FP80	40S ribosomal protein S8	3	− 1.56
A0T0C1	50S ribosomal protein L1, chloroplastic	7	− 1.66
A0T0C2	50S ribosomal protein L11, chloroplastic	6	− 1.45
A0T0C0	50S ribosomal protein L12, chloroplastic	17	− 1.69
A0T0K1	50S ribosomal protein L13	2	− 1.87
A0T0I9	50S ribosomal protein L14, chloroplastic	3	− 1.42
A0T0I6	50S ribosomal protein L16, chloroplastic	4	− 2.17
A0T0C7	50S ribosomal protein L19, chloroplastic	2	− 1.81
A0T0I1	50S ribosomal protein L2, chloroplastic	6	− 2.03
A0T0G3	50S ribosomal protein L21, chloroplastic	2	− 1.60
A0T0I4	50S ribosomal protein L22, chloroplastic	2	− 1.36
A0T0H8	50S ribosomal protein L3, chloroplastic	4	− 1.92
A0T0J3	50S ribosomal protein L6, chloroplastic	2	− 1.91
B7G9G2	60S ribosomal protein L13	7	− 1.34
B7G0R5	60S ribosomal protein L18a	10	− 1.77
B7FTL3	60S ribosomal protein L36	9	− 2.08
B7FUV3	60S ribosomal protein L6	7	− 1.48
E9PAI7	Elongation factor Ts, mitochondrial	6	− 1.36
B7GA11	Elongation factor Tu	10	− 1.62
B7G0T8	Eukaryotic translation initiation factor 3 subunit A	9	− 1.30
B7GCT6	Glutamyl-trna synthase	4	− 1.52
B5Y502	Ribosomal protein L15	6	− 1.62
B7GAA5	Ribosomal protein L19	7	− 1.49
	Protein processing		
A0T0H6	60 kDa chaperonin, chloroplastic	11	− 1.33
B7FUB7	ER luminal binding protein	33	− 1.45
B7G5I4	Importin subunit alpha	10	1.54
B7GE38	Oligosaccharyl transferase	3	− 1.26
B5Y4H4	Peptidyl-prolyl *cis*-trans isomerase	9	1.61
B7FQT3	Peptidyl-prolyl *cis*-trans isomerase	17	1.63
B7FSV6	Peptidyl-prolyl *cis*-trans isomerase	4	1.59
B7FPA6	Peptidyl-prolyl *cis*-trans isomerase	2	1.88
B7FZL3	Peptidyl-prolyl *cis*-trans isomerase	7	1.50
B7G5J3	Peptidyl-prolyl *cis*-trans isomerase	2	1.55
B7GB02	T-complex protein 1 subunit delta	3	− 1.28
	Proteolysis		
B7FU90	Proteasome subunit alpha type	2	− 1.29
B7G2F7	Regulatory proteasome non-atpase subunit 1	2	− 1.39
B7FY02	Ubiquitin extension protein 3	18	1.51
	Nitrogen Metabolism		
B7G8X8	Aliphatic amidase	2	2.24
B7GEG8	CPS III, carbamoyl-phosphate synthase mitochondrial	39	− 2.57
B7FYS6	Formidase	5	2.27
B7G997	Nitrate reductase	22	− 2.33
B7FZW5	Urea transporter	3	2.38
	Cytoskeleton/cellular transport		
B7G5C0	Actin/actin like protein	9	1.70
B7G878	Actin/actin like protein	23	1.42
B7FY56	Coronin	5	1.31
B7FTS7	Det3-like protein	7	1.54
B7FUJ2	Gelosin/severin like protein	6	2.42
	Histone		
B7FR39	Histone *H*3	8	1.29
B7FX68	Histone H4	12	− 2.13
B7FX66	Histone linker H1	6	1.51
B7FTP2	N-terminal histone linker H1	5	1.66
	Antioxidant		
B7G384	Ascorbate peroxidase	5	2.11
B7GDY5	Glutaredoxin	5	1.96
B7GDI2	Glyoxalase	2	1.80
B7G1J9	L-ascorbate peroxidase	6	1.50
B7G0L6	Superoxide dismutase	4	2.22
B7FP57	Thioredoxin	2	2.00
B7G0C9	Thioredoxin	5	2.43
B7G0P5	Thioredoxin f	3	1.31
B7G7L6	Thioredoxin h	3	2.44
	Heat shock protein		
Q41074	BiP	6	− 1.84
A0T0H7	Chaperone protein dnaK	33	− 1.40
B7FXQ8	Heat shock protein Hsp20	2	1.97
B7GEF7	Heat shock protein Hsp90	11	− 1.42
B7GCE9	Protein heat shock protein	10	− 1.42
	Miscellaneous		
B7G5Y2	14–3-3-like protein	11	1.64
B7FV10	1-Hydroxy-2-methyl-2-	7	− 1.70
B7S4B2	Alcohol dehydrogenase	2	3.30
A0T0F2	ATP-dependent zinc metalloprotease FtsH	11	− 1.75
B7FQH4	Calcyclin-binding protein	2	1.56
B7FNY6	Early light induced protein	3	− 1.95
B7FU89	Farnesyltranstransferase	5	− 2.13
B7GB73	FeS assembly protein suf	5	− 1.36
B7FUG8	Glycolate oxidase	10	− 1.50
B7FWY2	Hydroxymethylbilane synthase	22	− 1.84
B7FYL2	Iron starvation induced protein	6	− 3.90
A0T0E5	Iron-sulfur cluster formation ABC transporter ATP-binding subunit	5	− 1.36
B7G6D3	Metacaspase	5	1.69
B7S4C8	Methionine aminopeptidase	2	− 1.39
Q8LKV0	Microsomal cytochrome b5	3	− 1.81
B7FQ72	Mitochondria-targeted chaperonin	58	− 1.30
B7FU88	P2B, P type ATPase	3	− 1.28
B5Y5C8	Short-chain alcohol dehydrogenase with NAD or NADP as acceptor	7	1.65
B5Y3S6	Transaldolase	5	1.96
B7GEF3	Translocator of the inner chloroplast envelope membrane 110 k	13	− 1.63
