# Supplementary material for: Proteome response of Phaeodactylum tricornutum, during lipid accumulation induced by nitrogen depletion
Source: Algal Res. 2016 Sep;18:213–24. doi: 10.1016/j.algal.2016.06.015 (PMC5070409; doi:10.1016/j.algal.2016.06.015)

Supporting Information

Figure S1. Flow diagram outlining the processing of mass spectrometry data through six search engines to derives fold changes and statistical significance.


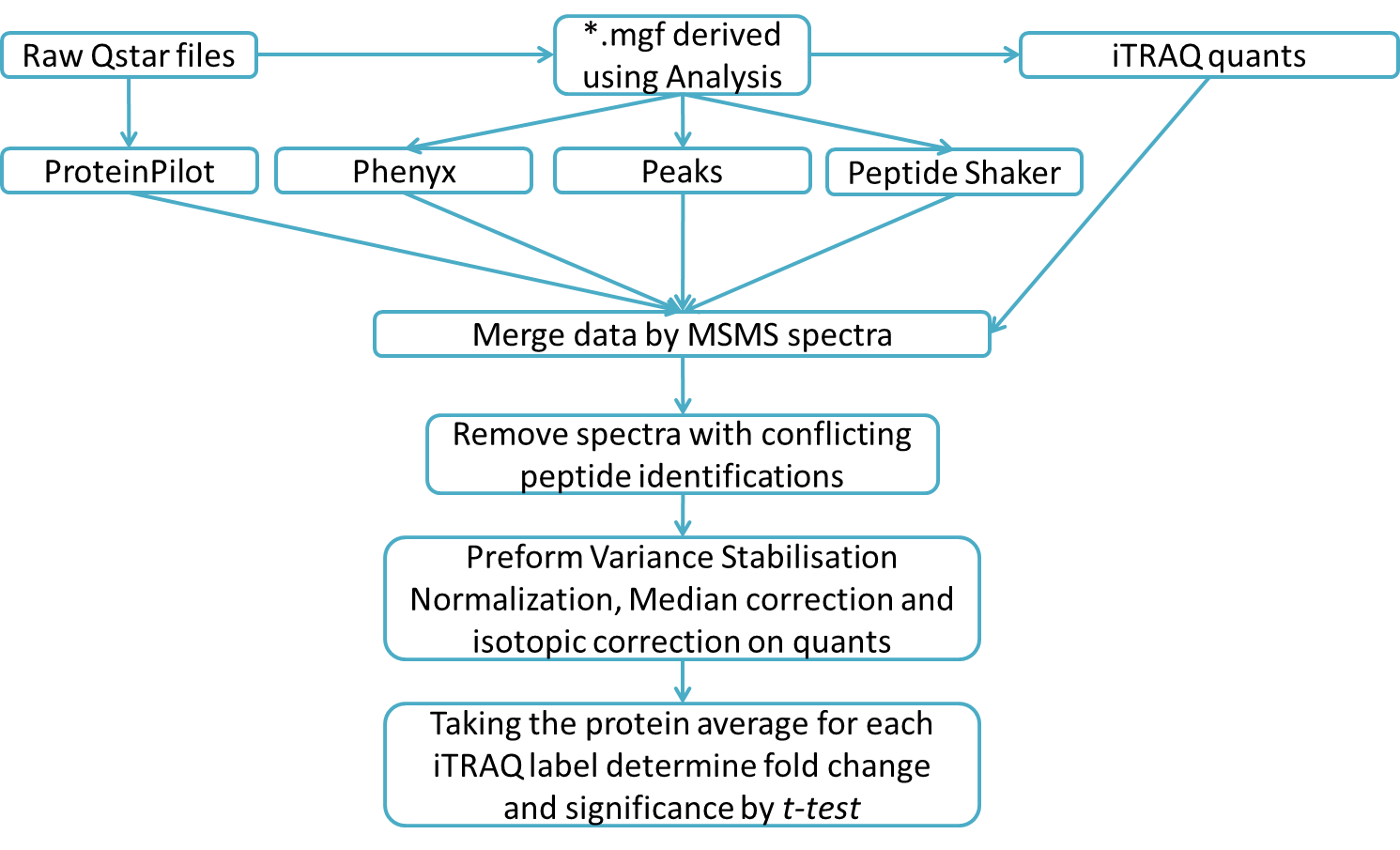


Figure S2. Plots showing the biochemical assessment of samples used for proteomic analysis. Samples taken 24h post change of media to test condition. Assessment of A) carbohydrate, B) lipid, C) chlorophyll A and D) total carotenoids are shown. All assessments were based on dry cell weight reconstituted to a fixed biomass concentration. *p*-values are reported indicating the significant difference of the two stress conditions to that of the normal replete condition.


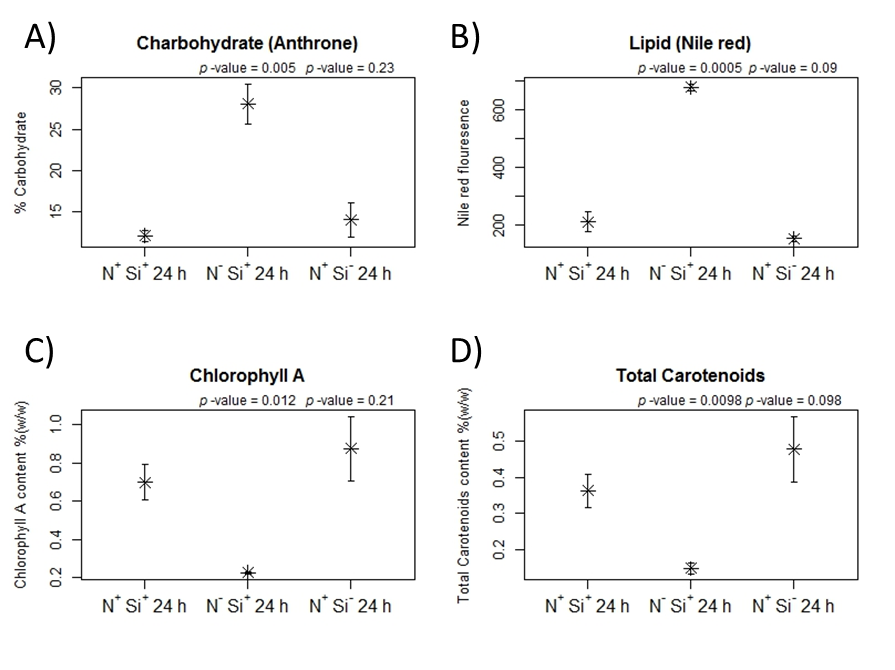


Figure S3. A) Dendrogram of the samples association with distance on y axis signifying degree of variation. A, B and C indicate normal, nitrogen stressed and silicon stressed respectively. B) Principal component analysis showing the clustering for samples based on VSN, IC, MC corrected reporter ion intensities. Limited to ≥2 peptide proteins.1, 2, 3 indicate biological replicates. Samples also indicate there label assignment 113:121.


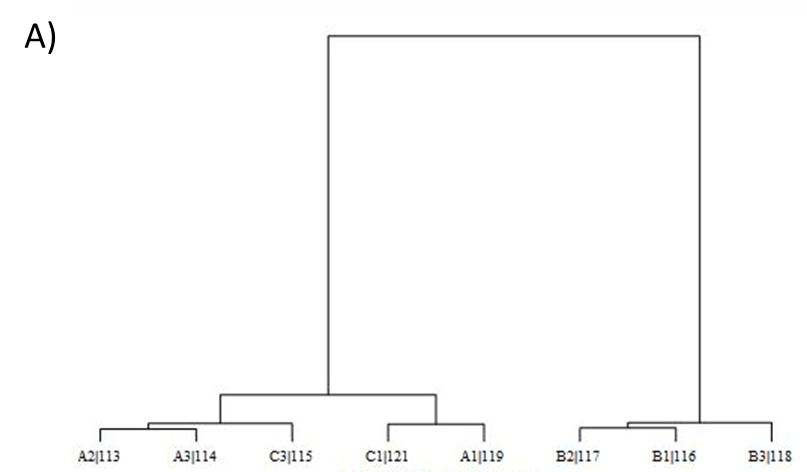

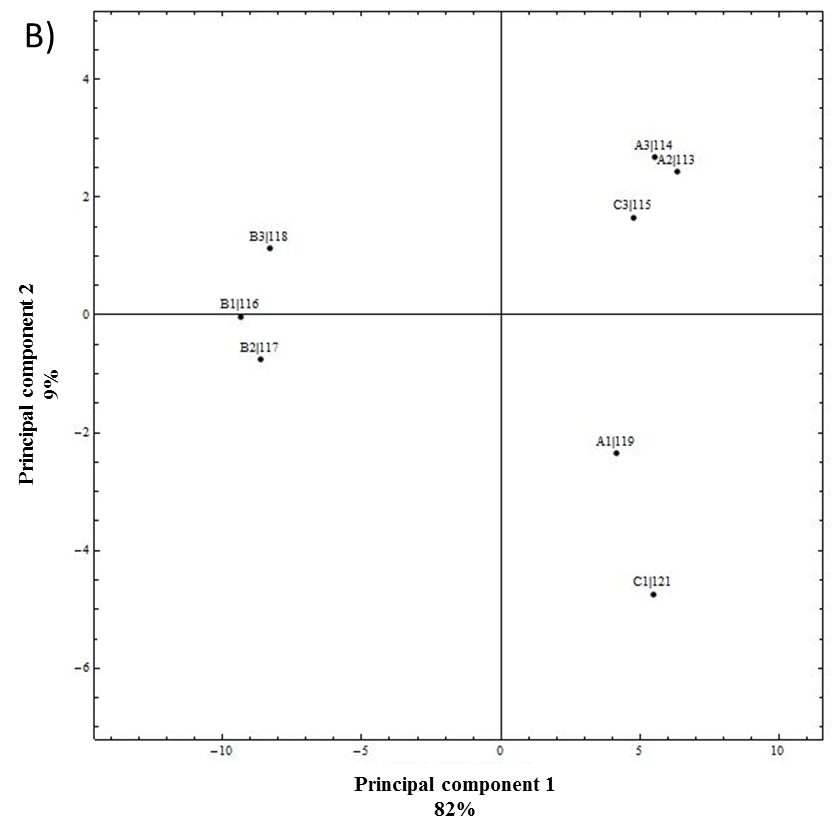


Figure S4. Gene ontology terms assignments indicating the number of protein assignments identified as significantly A) increased, B) decreased and C) both.


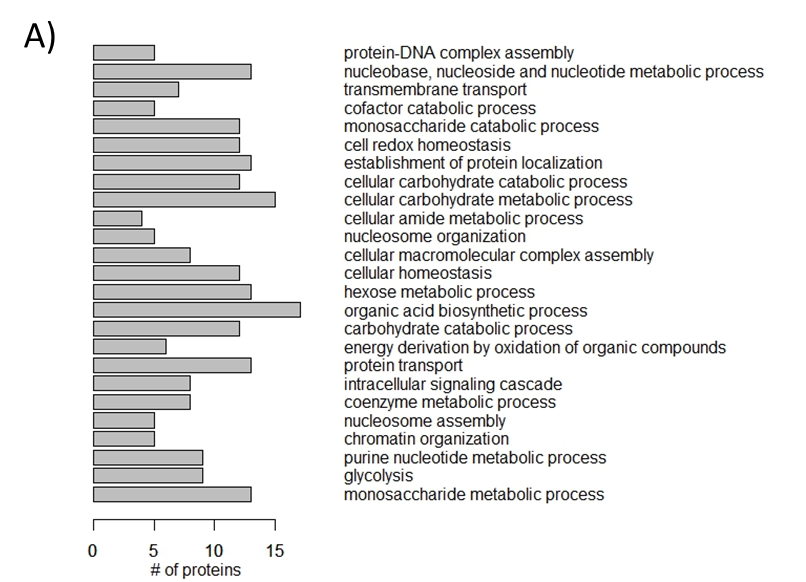


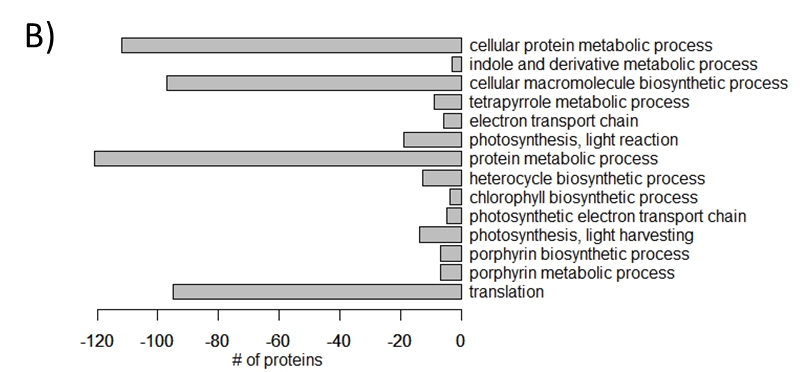


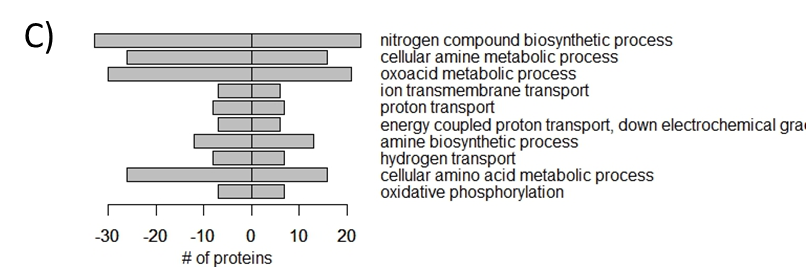


Figure S5. Ribosome diagram from KEGG painted with significantly (*p* < 0.05) regulated proteins. Increased abundance is shown in blue and decreased abundance shown in red.


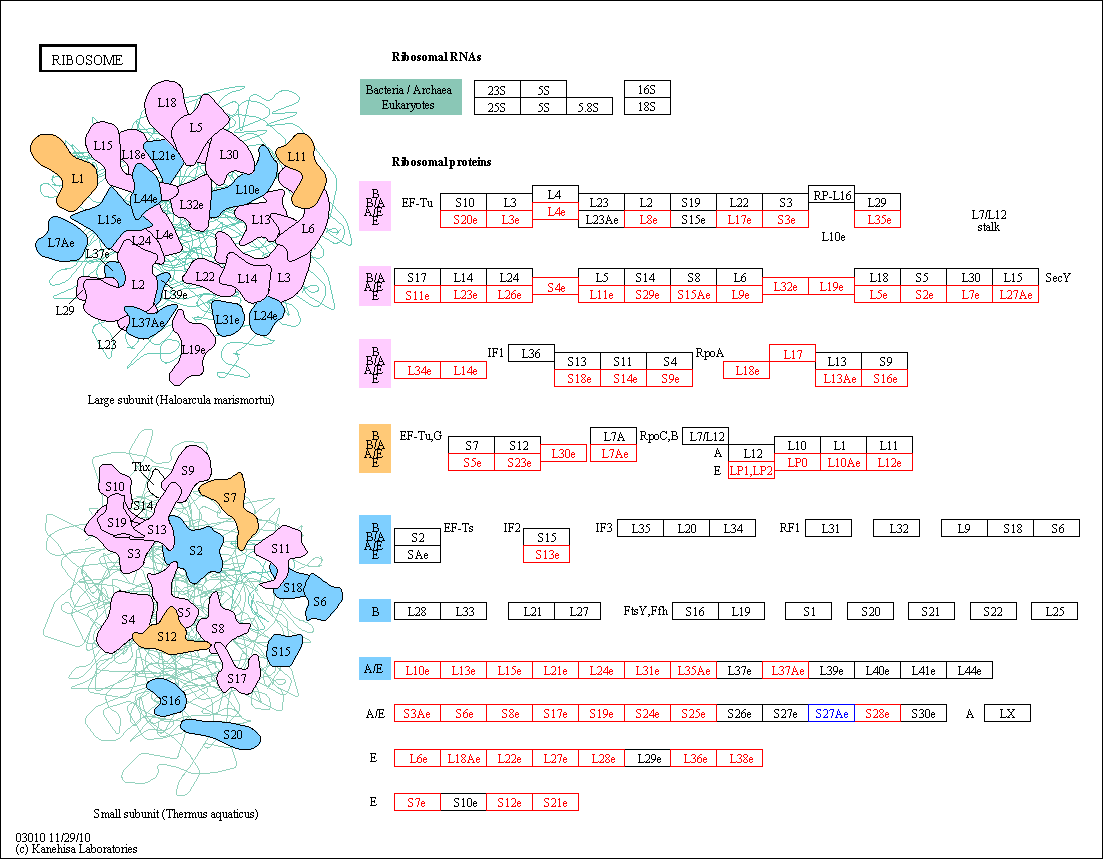


Figure S6. Amino acid production pathway diagrams from KEGG painted with significantly (*p* < 0.05) regulated proteins. Increased abundance is shown in blue and decreased abundance shown in red.


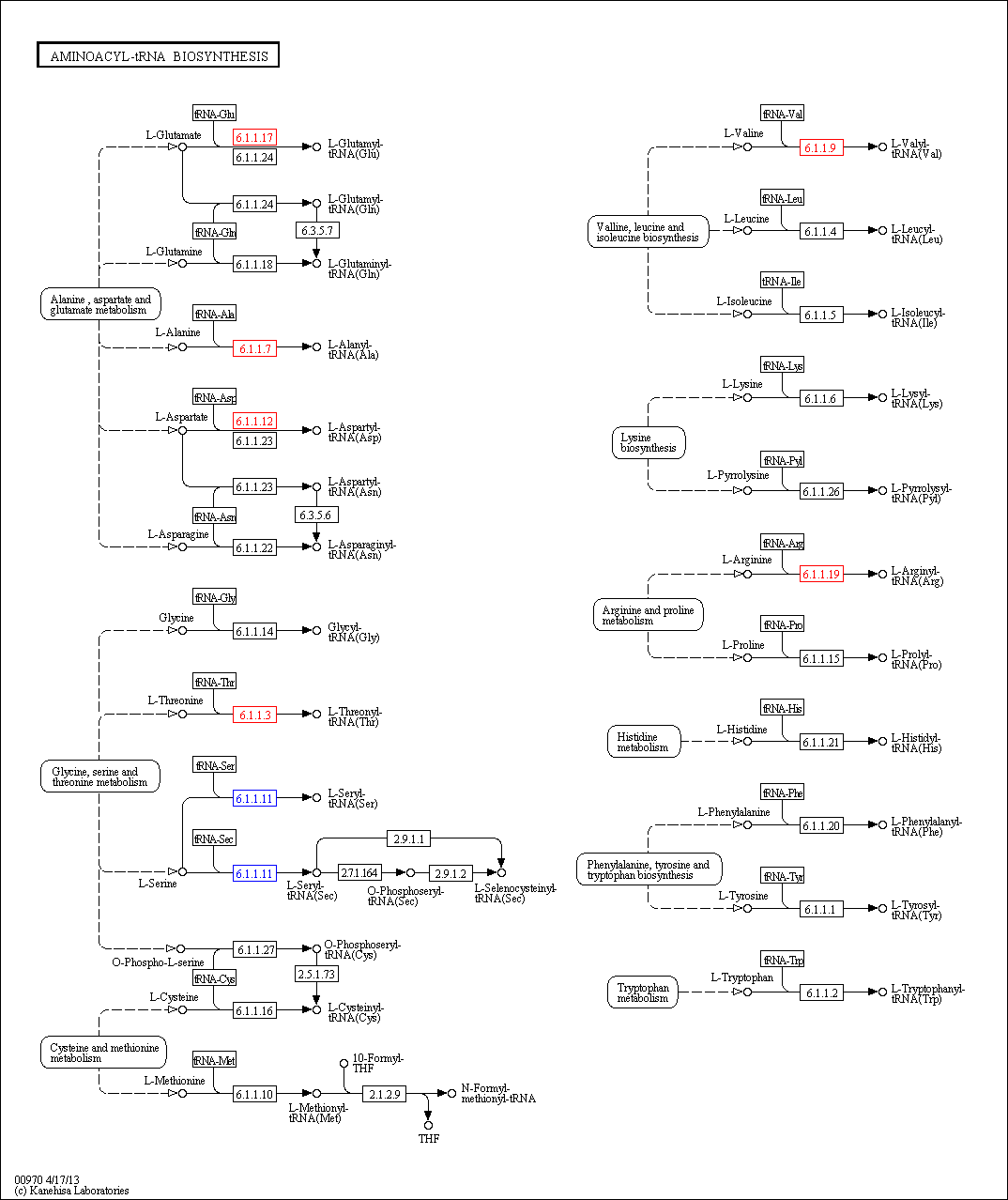


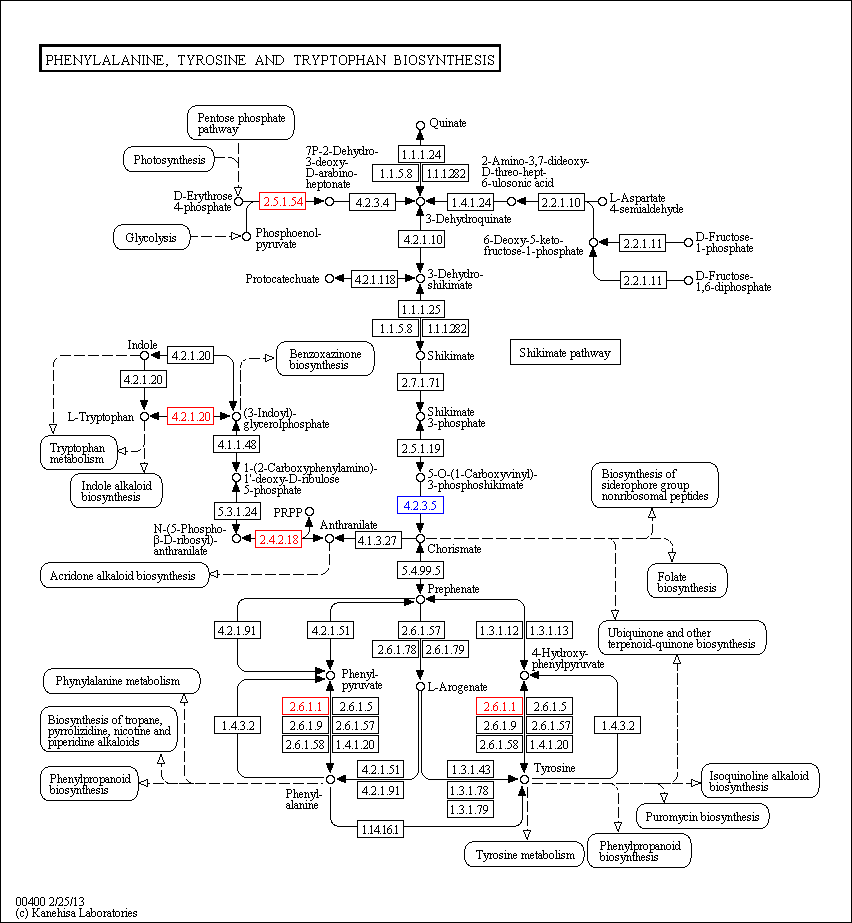


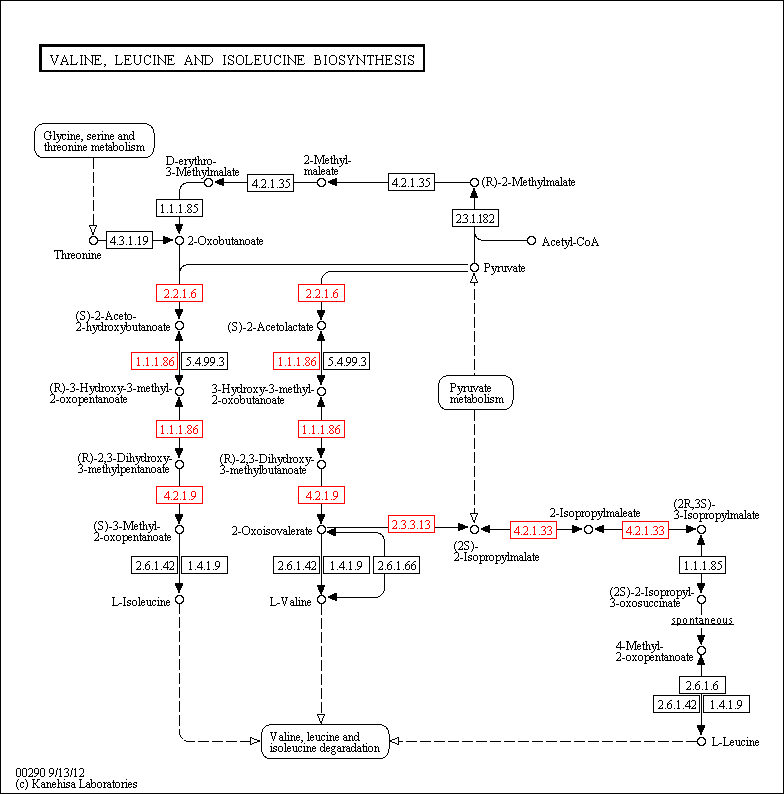


Figure S7. Central energy metabolism pathway diagrams from KEGG painted with significantly (*p* < 0.05) regulated proteins. Increased abundance is shown in blue and decreased abundance shown in red.


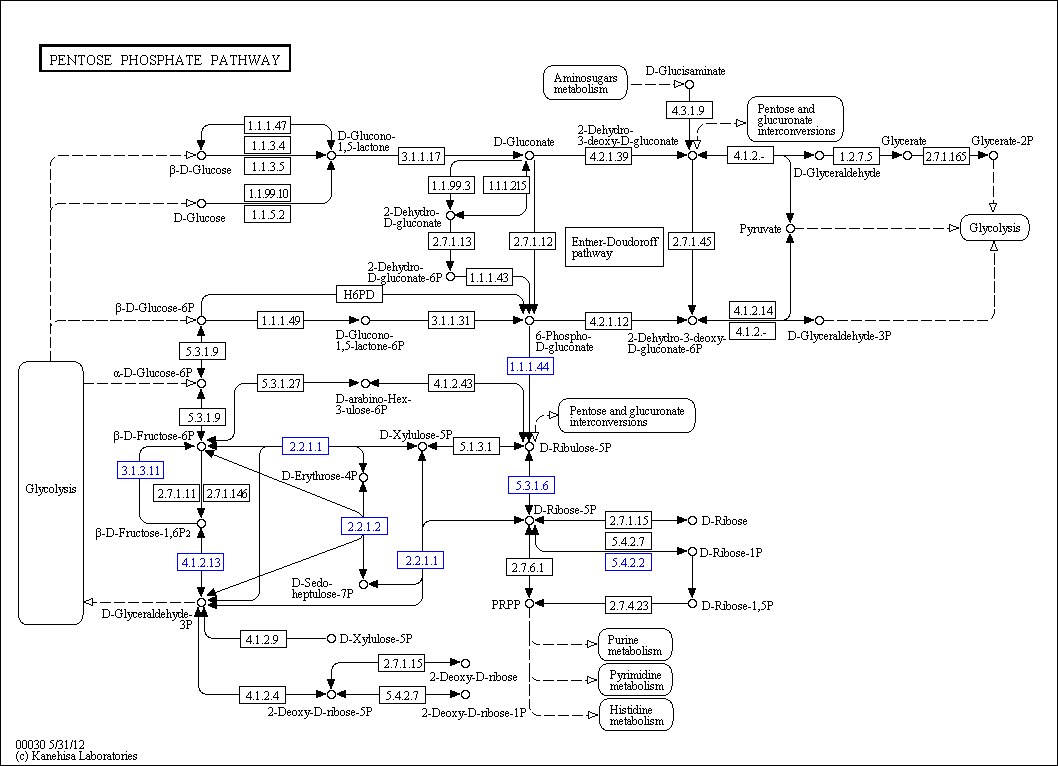

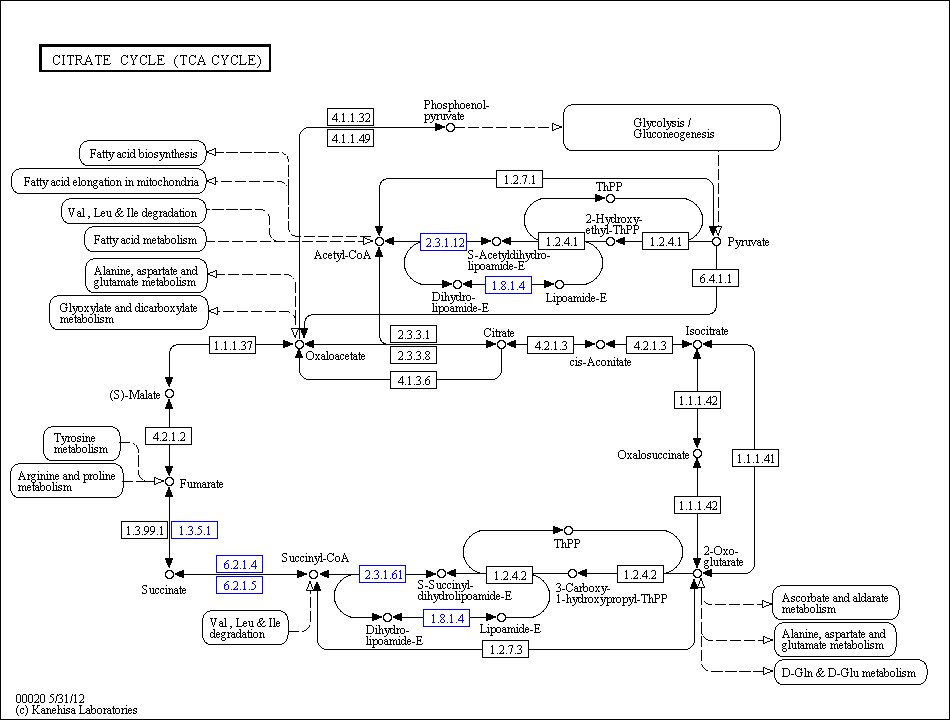


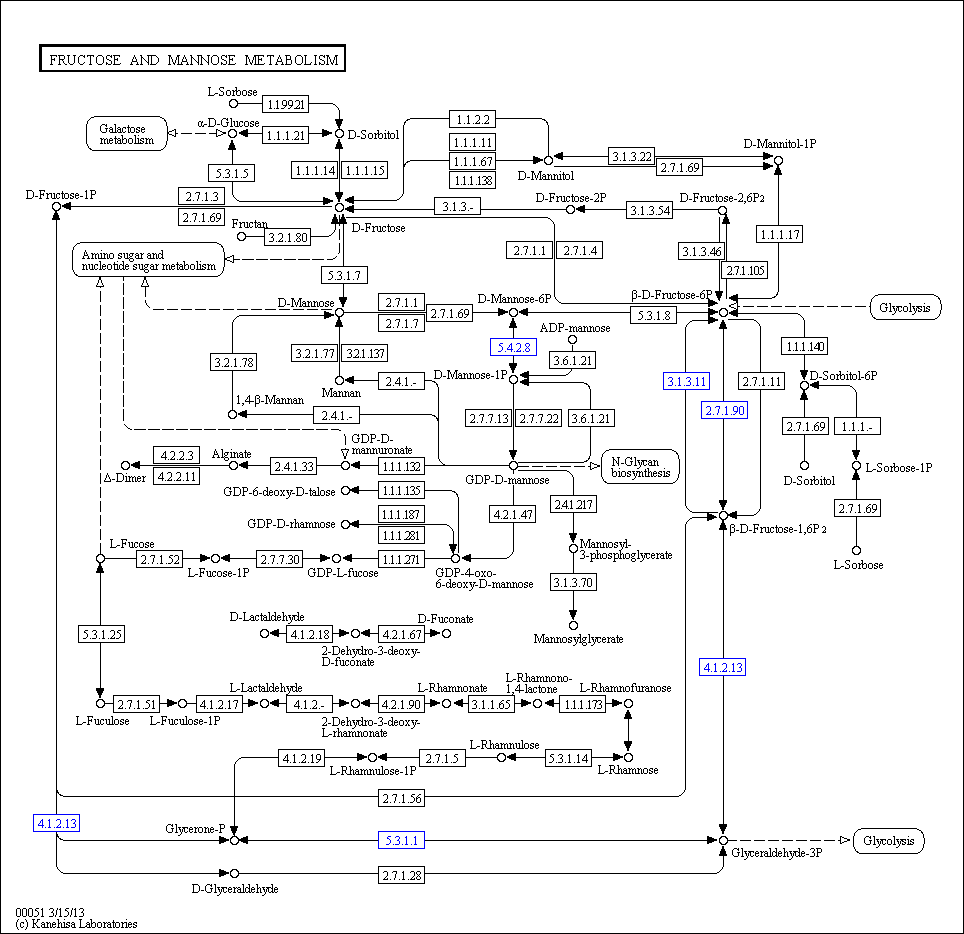


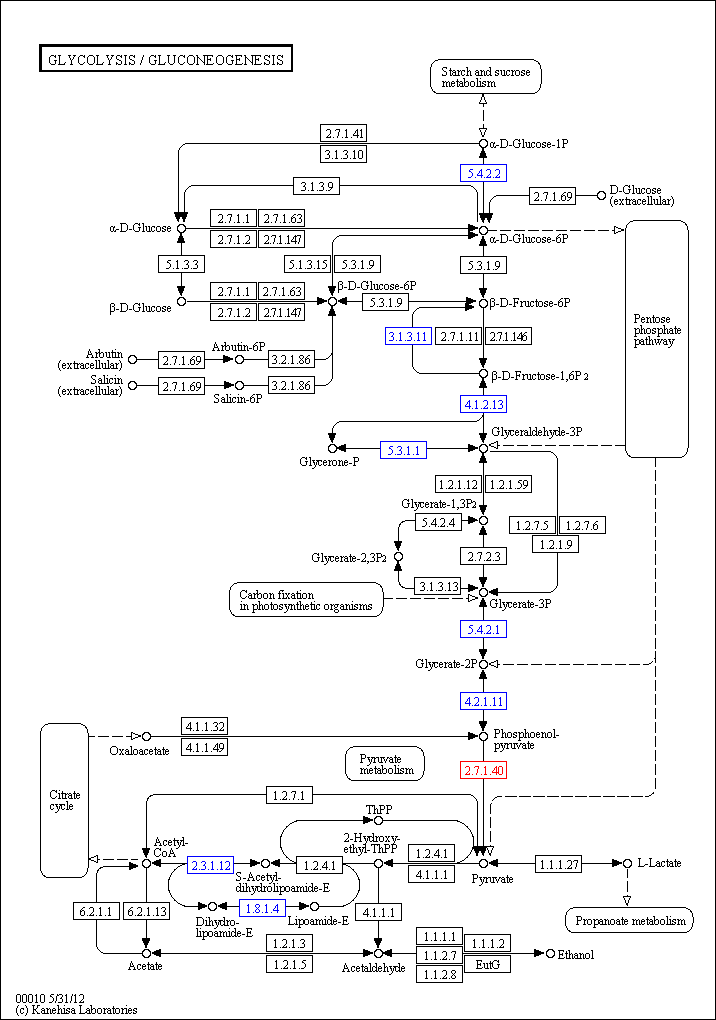


Figure S8. Photosynthetic apparatus diagram from KEGG painted with significantly (*p* < 0.05) regulated proteins. Increased abundance is shown in blue and decreased abundance shown in red.


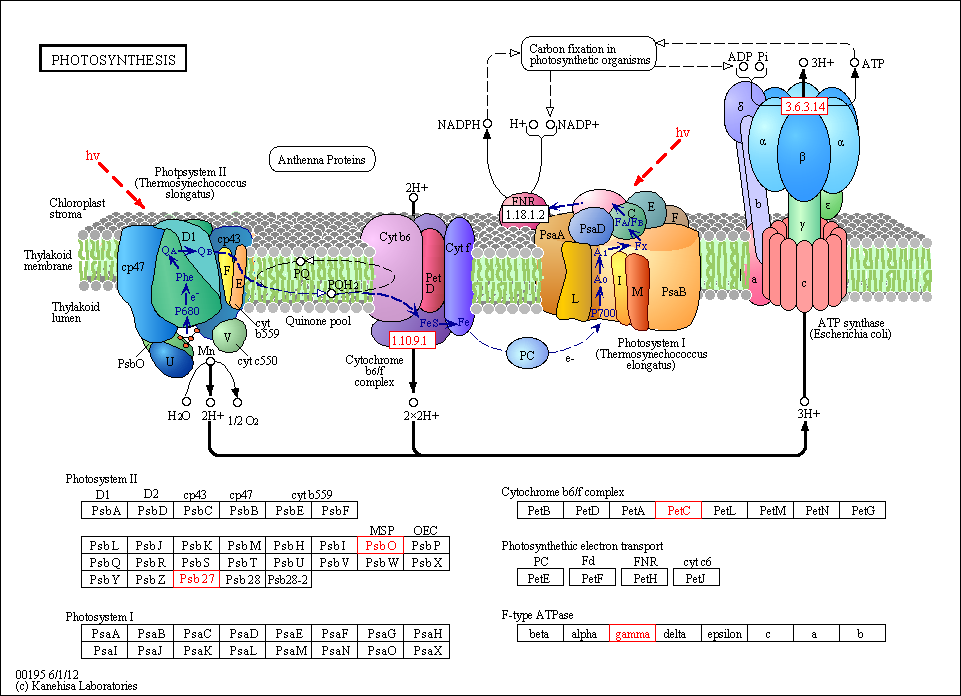


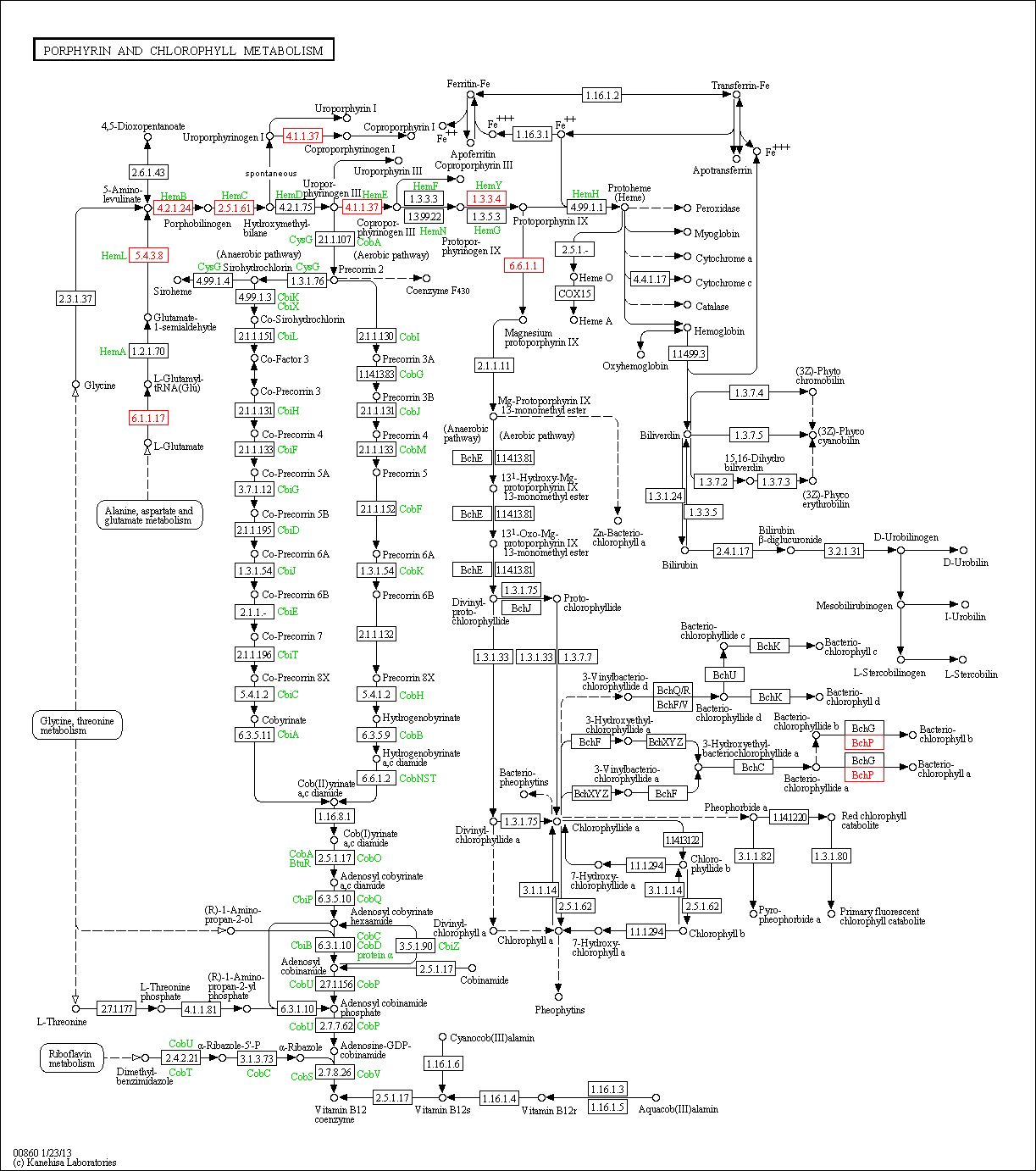


Figure S9. Fatty acid pathways diagrams from KEGG painted with significantly (*p* < 0.05) regulated proteins. Increased abundance is shown in blue and decreased abundance shown in red.


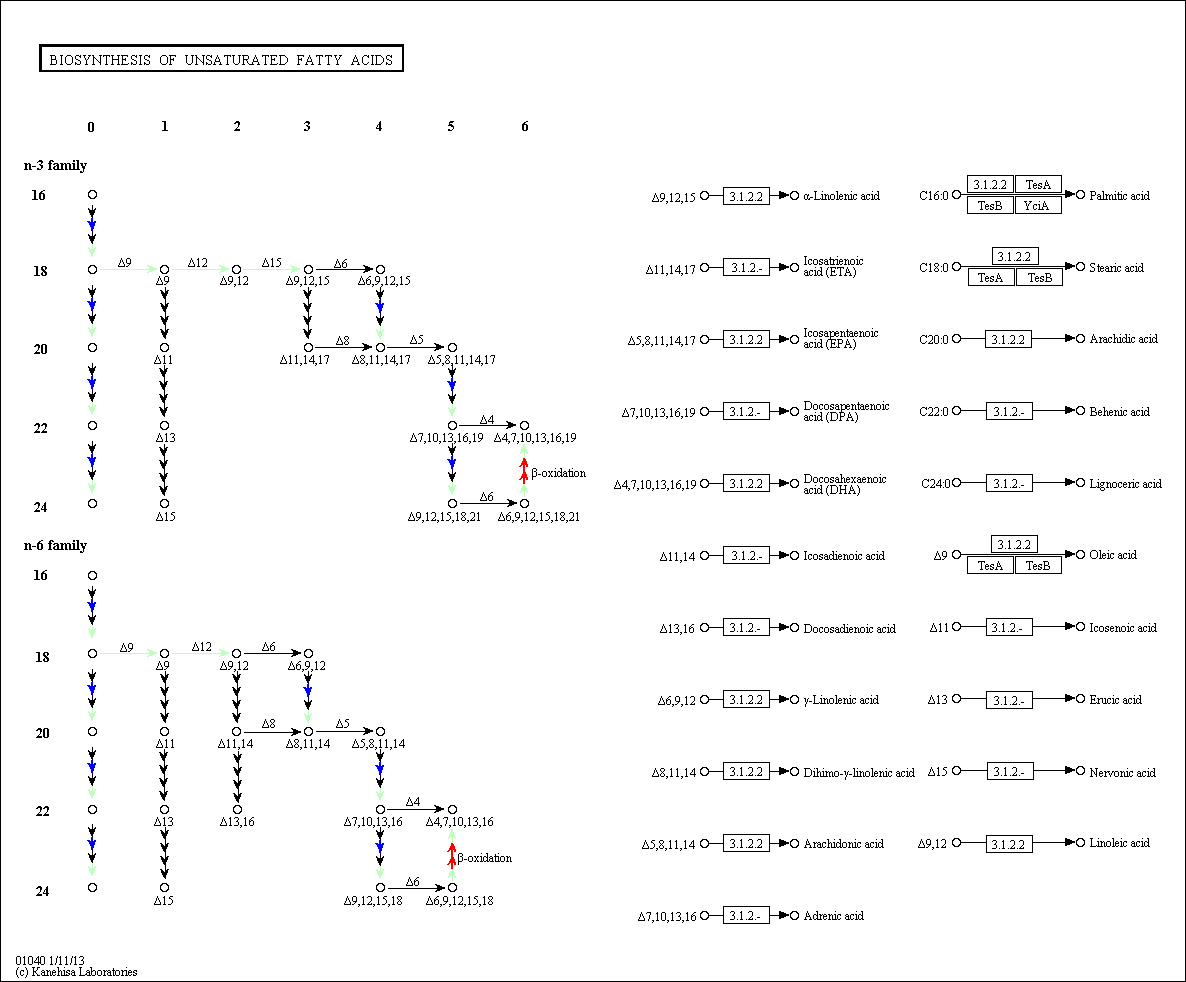


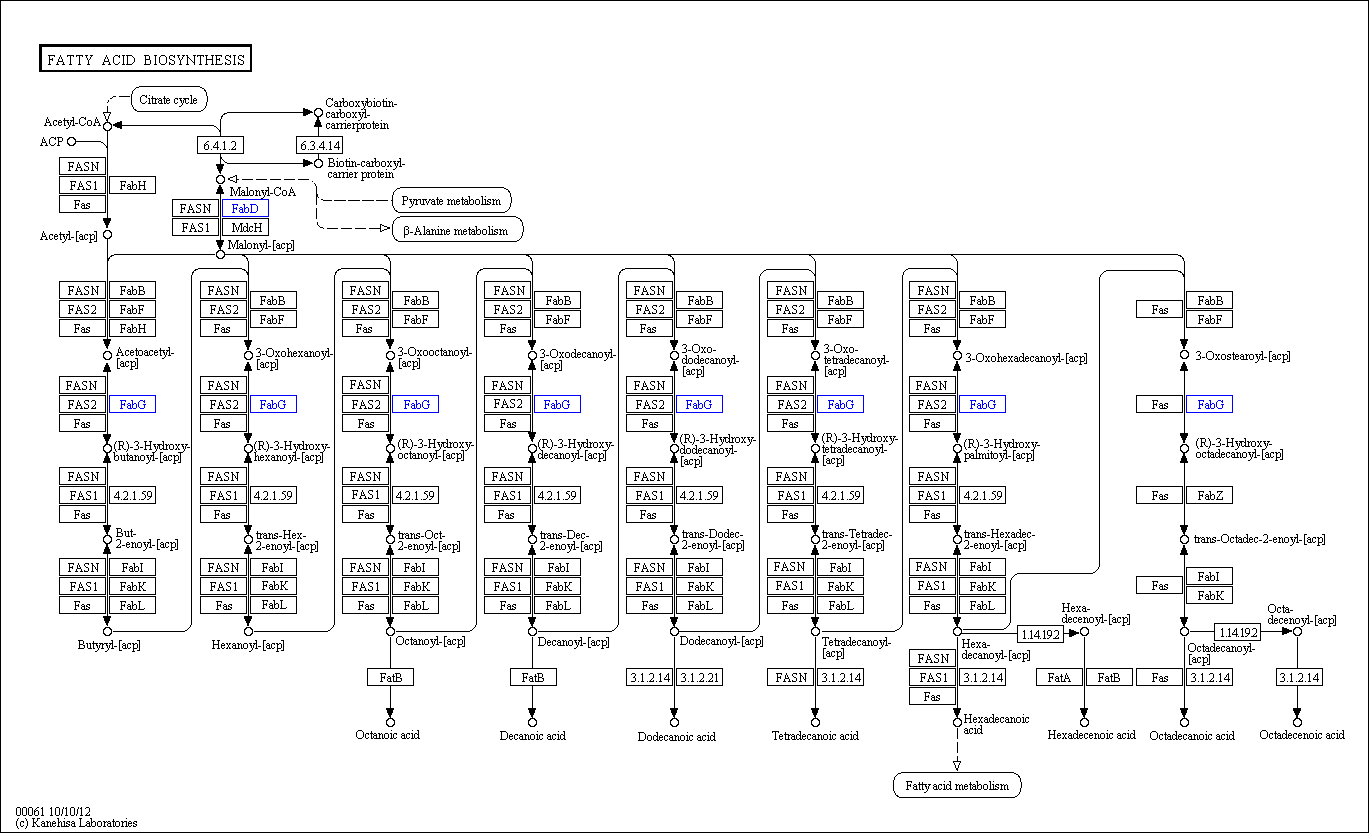


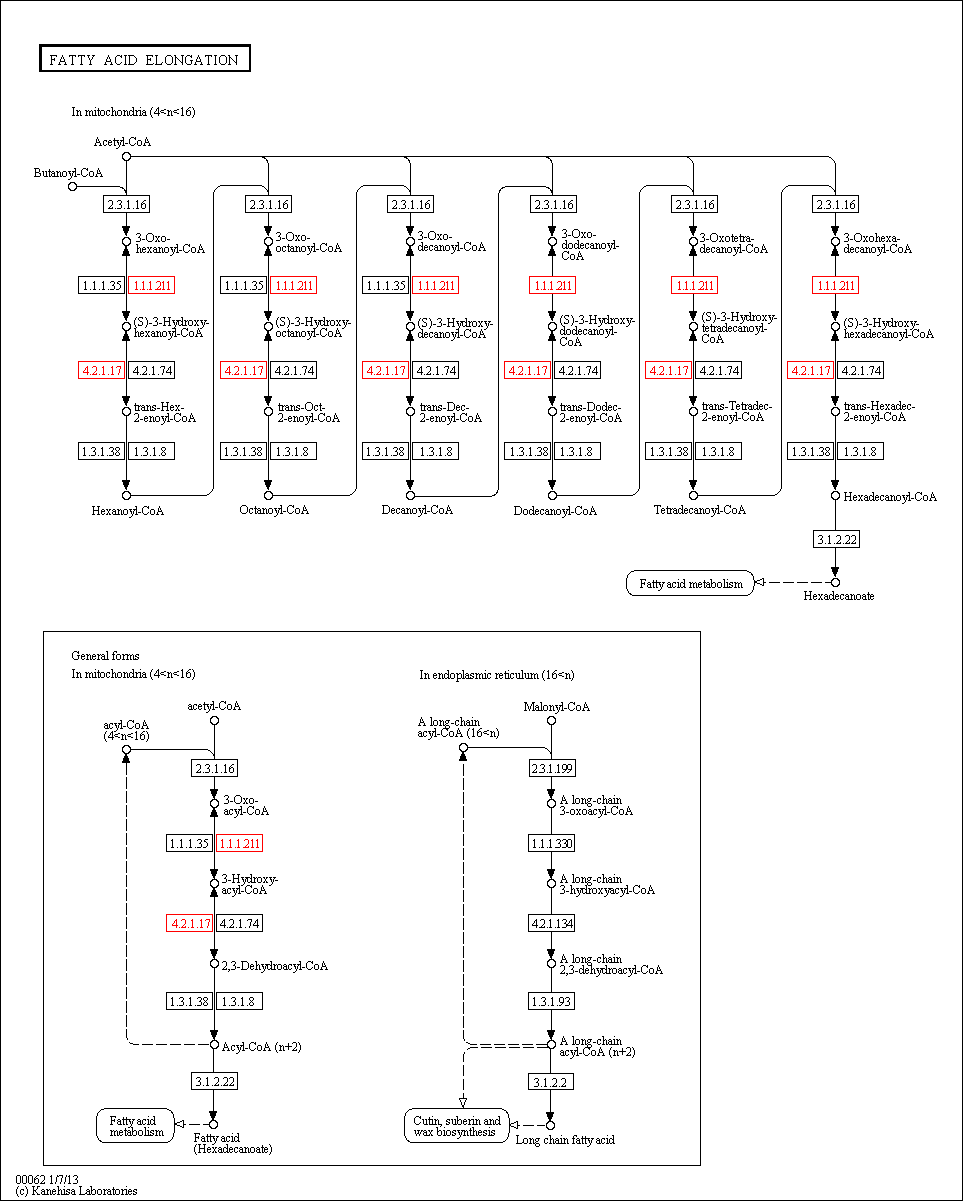


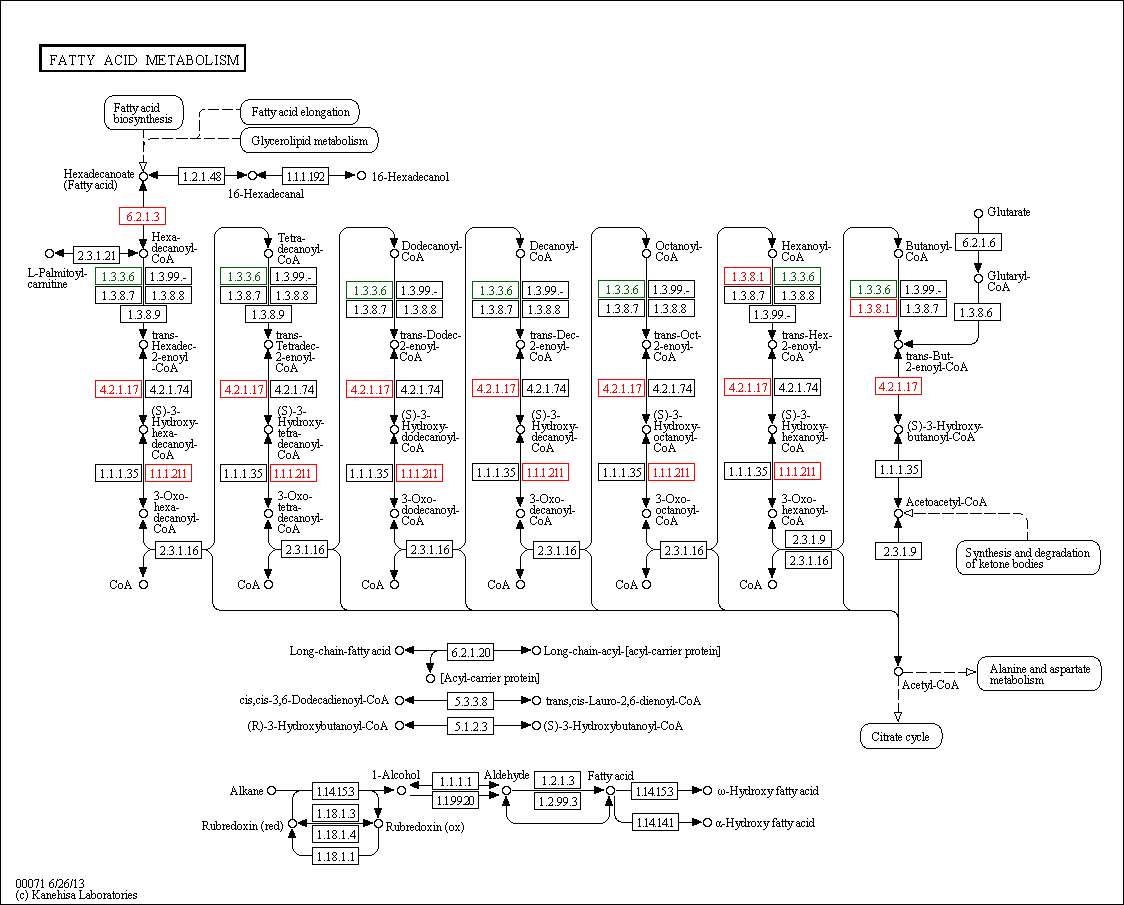


Figure S10. Endocytosis and Phagocytosis pathways diagrams from KEGG painted with significantly (*p* < 0.05) regulated proteins. Increased abundance is shown in blue and decreased abundance shown in red.


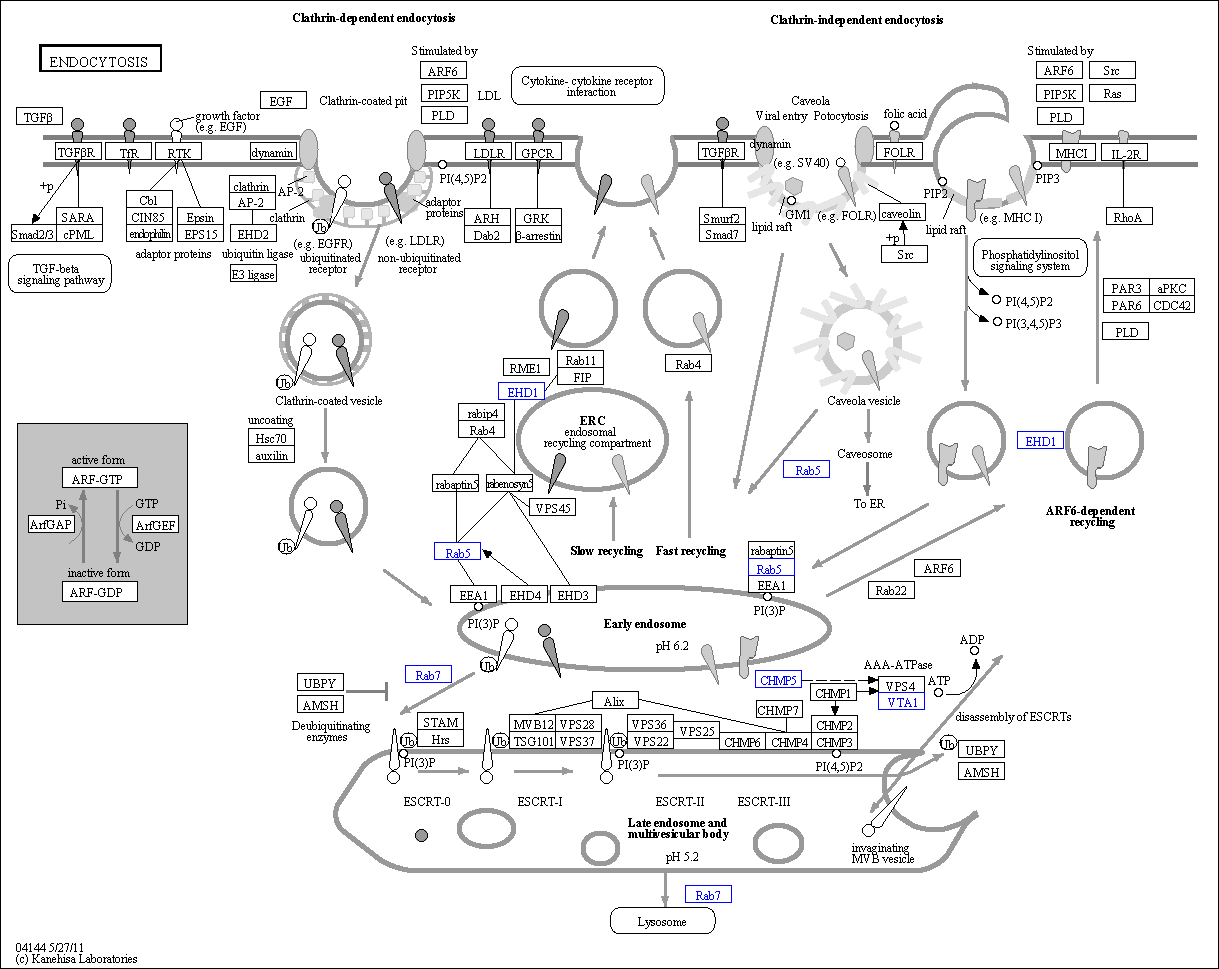


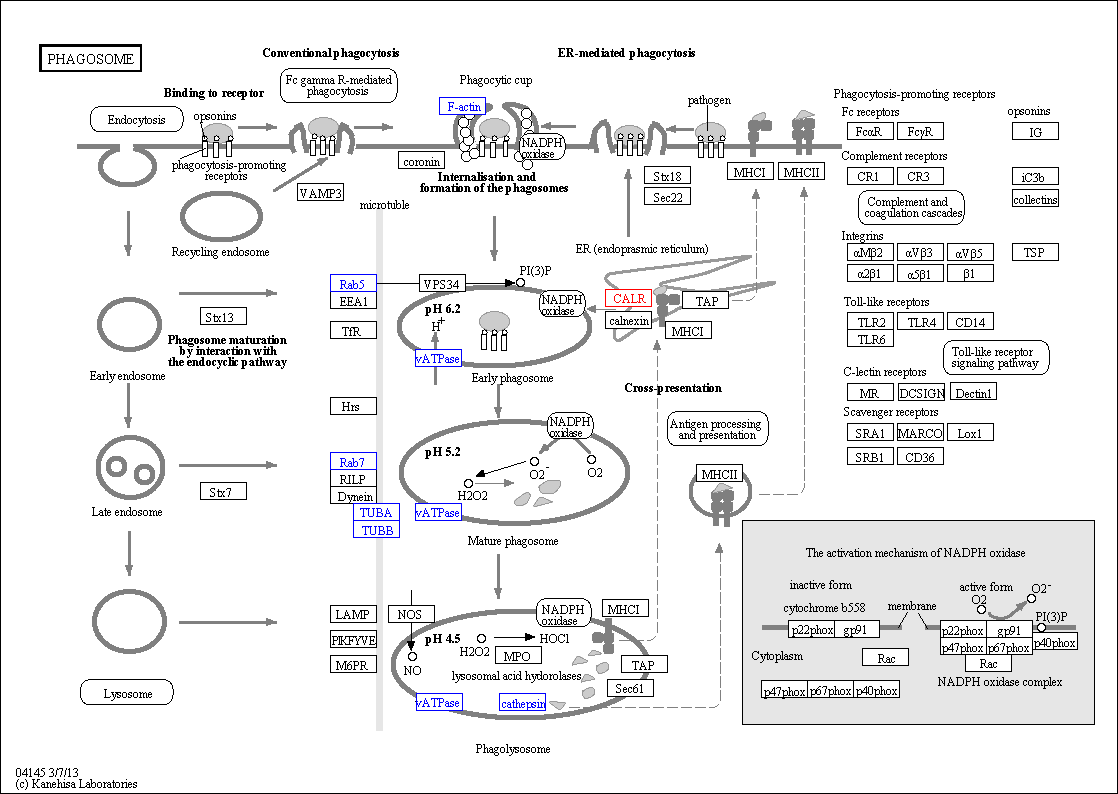

Supplement: Supplementary file 1 — Supplementary figures. [file mmc1.docx]
